# Ongoing human chromosome end extension revealed by analysis of BioNano and nanopore data

**DOI:** 10.1038/s41598-018-34774-0

**Published:** 2018-11-09

**Authors:** Haojing Shao, Chenxi Zhou, Minh Duc Cao, Lachlan J. M. Coin

**Affiliations:** Institute for Molecular Bioscience, University of Queensland, St Lucia, Brisbane, QLD 4072 Australia

## Abstract

The majority of human chromosome ends remain incompletely assembled due to their highly repetitive structure. In this study, we use BioNano data to anchor and extend chromosome ends from two European trios as well as two unrelated Asian genomes. At least 11 BioNano assembled chromosome ends are structurally divergent from the reference genome, including both missing sequence and extensions. These extensions are heritable and in some cases divergent between Asian and European samples. Six out of nine predicted extension sequences from NA12878 can be confirmed and filled by nanopore data. We identify two multi-kilobase sequence families both enriched more than 100-fold in extension sequence (p-values < 1e-5) whose origins can be traced to interstitial sequence on ancestral primate chromosome 7. Extensive sub-telomeric duplication of these families has occurred in the human lineage subsequent to divergence from chimpanzees.

## Introduction

The genome sequence of chromosome ends in the reference human genome remains incompletely assembled. In the latest draft of the human genome^1^ 21 out of 48 chromosome ends were incomplete; amongst which five chromosome ends (13p, 14p, 15p, 21p, 22p) are completely unknown and the remaining chromosome ends are capped with 10–110 kb of unknown sequence. There are many interesting observations in the chromosome end regions which remain unexplained, such as the observed increase in genetic divergence between Chimpanzee and Humans towards the chromosome ends^2^.

Chromosome ends contain telomere sequences and subtelomeric regions. Most human chromosome subtelomeric regions are duplications of other chromosome subtelomeric regions arranged in different combinations, referred to as subtelomeric duplications (STD). STD are highly divergent between species or even different populations of the same species^3,4^ and have experienced rapid adaptive selection^3^. Subtelomere length polymorphism is also found in humans^5^. The majority of subtelomeric duplications have the same orientation towards the chromosome end^3,4^. Based on this it has been suggested that they originated from reciprocal translocation of chromosome tips and unbalanced selection^4^.

Telomere repeat sequences ([TAAGGG]n) - which are the capping sequences of chromosome ends - are breakable, acquirable and fusible in the genome. In somatic cells, telomeres are observed to progressively shorten^6,7^. If the telomere sequence is lost, the broken chromosome will become unstable^8–10^, and multiple types of rearrangements can occur, including chromosome fusion^8^, tips translocation^11^, or direct addition of telomere repeats^10^. Manual insertion of telomere repeat sequence in the interstitial region results in enhanced chromosome breakages and induces high rates of chromosome rearrangements around the insertion^12^. Interstitial telomeric sequences (ITS) are widespread in the genome^13–15^. In subtelomeric regions, they are almost always oriented towards the terminal end of the chromosome, like the STD^4^.

The quality of assembly for the chromosome ends largely depends on the sequencing technology. To correctly assemble highly duplicated regions like chromosome ends, sequence reads or read pairs spanning the repeat are required^16^. Recently, the NanoChannel Array (Irys System) from BioNano Genomics^17^ was introduced. This technology can generate barcodes on DNA fragments which are hundreds of kilobases long by detecting the distance between specific enzyme recognition sites. Alignment and genome assembly are performed based on numerous distinct site distance fragments. These very long fragments enable construction of individual physical maps, as well as completing the reference in unknown regions^18^. In this manuscript, we report on observed subtelomeric dynamics using this technology for the first time. We conclude that these genome dynamics reflect ongoing chromosome extension and deletion and identify genomic regions which have undergone substantial extension in multiple primate lineages.

## Results

### Assembling chromosome ends using BioNano data

**Figure 1:**
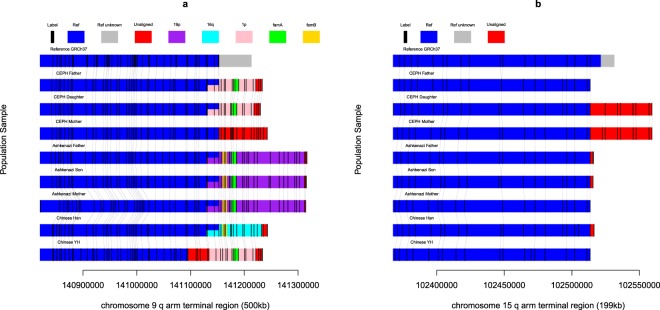
Paralogy map for chromosome tips region. (**a**) 9q; (**b**) 15q. These chromosome tips are assembled by BioNano data from two trios and two individuals. The enzyme recognition sites (labels) are marked as black bars and gray connecting lines indicate alignment between samples. The homologous sequence is indicated by color block: blue indicates homology to human reference sequence at the given chromosome; grey indicates unknown reference sequence; purple, cyan and pink indicate homology to 19p, 16q and 1p respectively. The remaining unaligned regions are all colored with red. Overlapping colors indicate a shared homology to multiple sources. Yellow and bright green indicate homology with family A and family B respectively. An overlapping color indicates this region is homologous to two regions.

We downloaded data from BioNano Genomics (see Methods). The data contains eight samples, including two family trios (Ashkenazi, CEPH) and two Chinese samples. The raw data has been assembled into contigs (with N50 of 3.3 MB) and aligned to the GRCh37 reference. We used the most distal unique aligned sequences at the chromosome ends to anchor individual chromosome ends (see Methods, Figure S1, Table S1–4 and Fig. 1). We also evaluated this alignment by OMBlast^19^. 92% of the terminal contig is still aligned at the same terminus. 7% of the terminal contig is unaligned at all with mean size 1.8 Mb. We regard Chromosome X and Y as the same chromosome for this analysis because their ends are homologous, namely the pseudoautosomal region (PAR). We removed 10 termini from the analysis because of heterochromatin (13p, 14p, 15p, 21p and 22p) and reference gaps (1p, 2q, 12p, 17p and XYp). Given the low resolution of Bionano (the mean inter-label distance is 8.2 kb), we use a threshold of 33.1 kb, equal to the average Bionano inter-label distance + 3* standard deviations to filter identified missing and extension terminal sequence. Of remaining 36 termini, 21 have no extension or missing sequence greater than threshold in any of 8 samples (1q, 2p, 3p, 4p, 5q, 5p, 6q, 8p, 8q, 9p, 10p, 11q, 12q, 13q, 16q, 17q, 18q, 18p, 19p, 21q and XYq, Table S1 and S2). 4 termini (7q, 14q, 19q and 22q) had only missing terminal sequence but no extension. The presence of missing terminal sequence could be due to misassembly or misalignment. Thus, we focus on extended termini. We identified extensions in 11 termini relative to GRCh37. We observed a consistent extension combined with reference deletion at 2q terminus which supports the re-orientation of chromosome 2q ends in the new GRCh38 reference (Figure S2a). We also observed extension sequence at 9q in the Ashkenazi family which matched the GRCh38 extension of this chromosome (Figure S2b). We also observed a 260 kb extension sequence in one sample at the 16p terminus which was originally discovered in 1991^5^ (Figure S1.31).

We classify the observed chromosome end extensions in 11 termini as category 1 or 2 based on whether the extension BioNano label sequence can be aligned to homologous sequence or not (see Methods). For example, Fig. 1a indicates an extension of 9q using the terminus of 1p (referred to as 9q-to-1p), such that the distal 20 Kb of 9q is homologous to the proximal 20 Kb of 1p. We observed 30/64 terminal extensions which fall into category 1, including 3q-to-19p, 6p-to-5q, 7p-to-1p, 11p-to-19p and 11p-to-5q, in addition to the 9q-to-1p extension. The remaining 34 individual chromosome end extensions fall into category two, including extensions to 4q, 6p, 7p, 9q, 10q, 11p, 15q, however, we note that inability to recognize homology may be caused by the low resolution of BioNano. The 260 kb 16p extension includes homology to both 3q and 19p.

### Discovery of heritable chromosome end polymorphism

We revealed characteristics of chromosome ends by analysis of completed chromosome ends of the eight samples. In the extension termini, three out of eleven (3q, 7p and 20q) show almost identical extension sequence across all eight samples, while the remaining eight out of eleven show population polymorphism. For example, four types of extension sequences (unaligned, 1p, 19p and 16q) are observed in 9q arm amongst 8 samples (Fig. 1a). A 45 kb extension sequence is observed only in CEPH mother and daughter in 15q (Fig. 1b). We observed that the daughter chromosome end sequences (both missing and extending sequence) are almost always (93.8%, 75/80, see Methods) also observed in one of her parents. The heritability of chromosome end extension indicates that the chromosome end data are unlikely to be a result of an assembly artefact. Although it is technically challenging to recreate the diploid terminal sequence from the BioNano data, we manage to identify heterozygous chromosome ends at 4q for two trios (see Methods, Figure S3). In this case, a diploid analysis was able to reveal a heterozygous chromosome end extension in the daughter, with the extended sequence inherited from the father, and the non-extended sequence inherited from the mother.

### Validating and filling chromosome end extension by nanopore data

**Figure 2:**
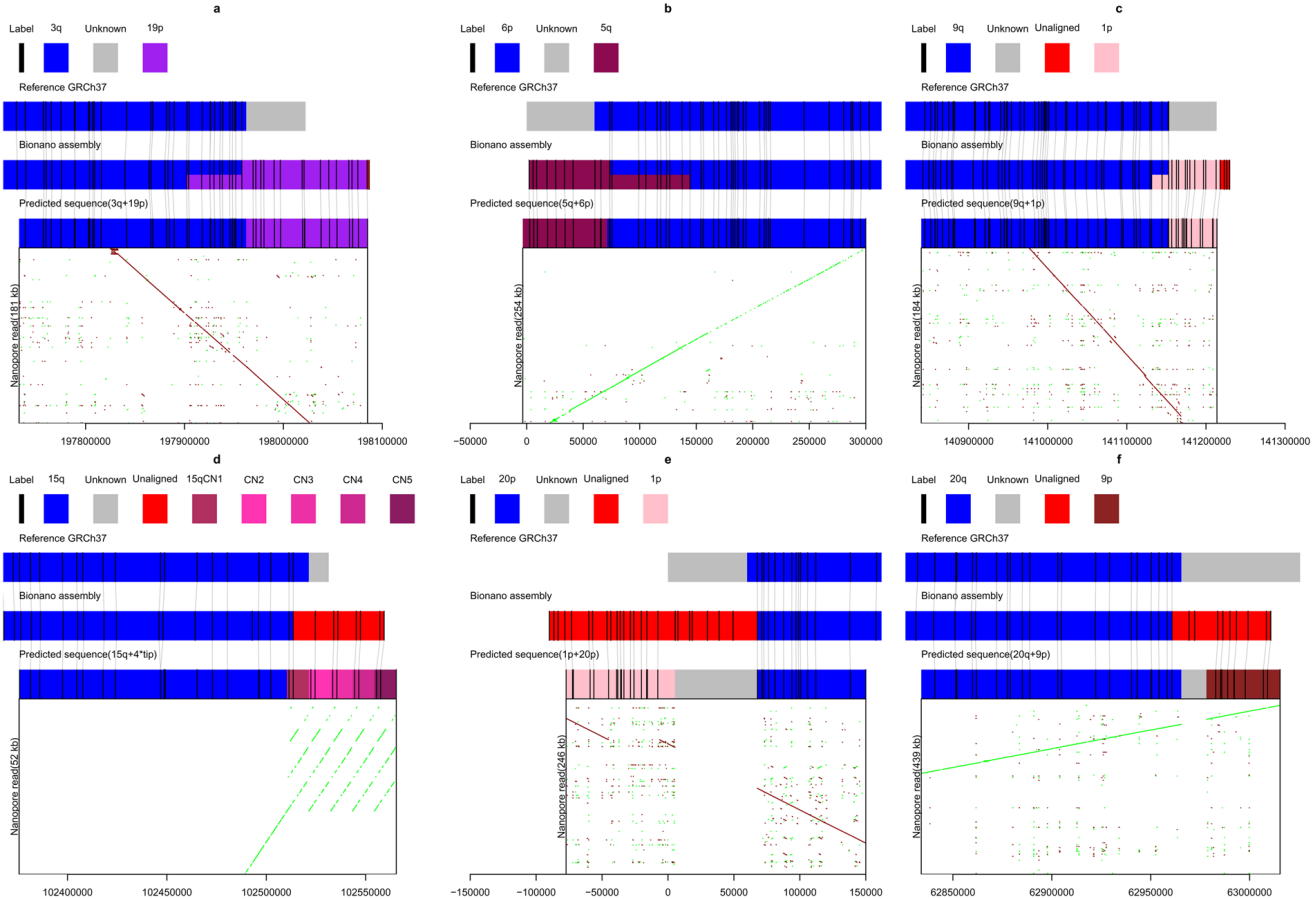
Validation of six termini by nanopore reads. (**a**) 3q; (**b**) 6p; (**c**) 9q; (**d**) 15q; (**e**) 20p; (**f**) 20q. Reference, bionano assembly in NA12878 and predicted extension sequence (see Methods) are showed as coloured rectangle in the middle. In silico bionano enzyme recognition sites (labels) are showed as vertical black line. The grey lines between labels indicate they are matched. The dotplots of nanopore read to extension sequence are shown at the bottom. Green and red are forward and reverse alignment, respectively. The nanopore read names are listed in Table S10.

The recent availability of ultra long nanopore sequence reads (>100 kb) from NA12878 (see Methods) allowed for orthogonal validation of extension sequence. We identified 9 confident chromosome extension events identified from BioNano analysis on NA12878 based on a minimum length threshold of 33 kb (Table S2). 6 of these were classified as category 1, such that the Bionano analysis was able to identify reference genome sequence homologous to the extension sequence which could be then used to construct predicted chimeric chromosome ends (Table S5). We then searched for nanopore reads which mapped to these chimeric chromosome ends, which was successful for 3q-to-19p, 6p-to-5q, 9q-to-1p, 20p-to-1p (Fig. 2a–c,e). These chimeric sequences were the best alignments for these reads. For the predicted extension on 20p, the nanopore alignment contained a large internal deletion, indicating that the extension was more complicated than simple extension via duplication of 1p (Fig. 2e). The 11p and 5q chromosome ends share a 106 kb overlap (Table S5), and moreover are highly homologous to the predicted 3q-to-19p chimeric sequence and as a result, all the nanopore reads which mapped to 11p-to-5q chimera mapped equally well to 11p or 5q separately. Although we identified long nanopore reads which mapped to 7p-to-1p chimera, only a small proportion of this alignment matched the 1p extension sequence (Figure S4a). For the remaining predicted category 2 extension sequences, we were unable to identify regions matching the extension sequence from Bionano analysis and pursued an alternative strategy of searching for the longest nanopore read mapping to the chromosome end. This enabled identification of the chromosome end extension sequence for 15q as being a 4 times duplication of 11 kb of its own terminal sequence (Fig. 2d). Notably, The distal part of 15q is capped with proper telomere in the reference, and this telomeric sequence is part of the 11 kb repeat in the nanopore reads (Figure S5). This approach also resolved the extension sequence of 20q as being homologous to 9p (Fig. 2f). Analysis of nanopore reads mapping to the terminus of 4q revealed homology to its own terminus, indicating another potential tandem repeat. However, this homology was not contiguous, indicating duplication followed by substantial structural variation (Figure S4b).

### Inferring the origin of extension sequences

**Figure 3:**
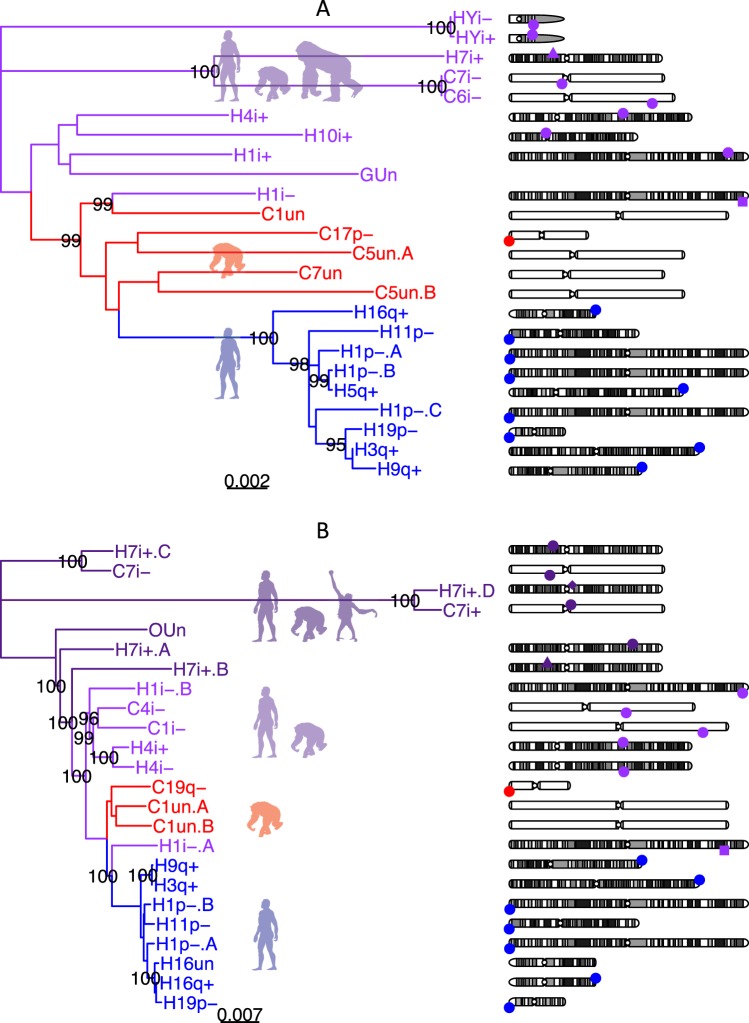
Phylogenetic trees for two sequence families identified in human extension sequences. (**A**) Family A (9 kb); (**B**) Family B (8 kb). Phylogenetic trees are generated from their homologous sequences. Dark purple, light purple, red and blue indicate intra-chromosome duplication group, inter-chromosome duplication group, chimpanzee subtelomeric duplication group and human subtelomeric duplication group, respectively. H for Human, C for Chimpanzee, G for Gorilla and O for Orangutan. p for p arm, q for q arm, Un for unplaced contig, un for unlocalized within a chromosome and i for interstitial genome regions. ‘+‘ for forward aligned and ‘−’ for reverse aligned. ‘.A’, ‘.B’, ‘.C’ and ‘.D’ are appended to distinguish different copies in the same region. Bootstrap value more than 95 is shown. Their genome location is shown as different shapes at each chromosome on the right. The human ancestor copy of intra-chromosome duplication group, inter-chromosome duplication group, and subtelomeric group are indicated by the shape of a diamond, triangle, and square. Otherwise, the shape is a circle. Top and bottom indicate different orientation. Gene FAM157A, FAM157B and FAM157C contain both families A and B at H3q, H9q and H16q, respectively. Silhouette images are from PhyloPic. Homo sapiens (“http://phylopic.org/image/c089caae-43ef-4e4e-bf26-973dd4cb65c5”) and Gorilla (“http://phylopic.org/image/d9af529d-e426-4c7a-922a-562d57a7872e/”) are both licensed under the Public Domain Dedication 1.0 license. Pan troglodytes (“http://phylopic.org/image/2f7da8c8-897a-445e-b003-b3955ad08850/”) by T. Michael Keesey (vectorization) and Tony Hisgett (photography), and Pongo abelii (“http://phylopic.org/image/67144c22-93c2-4dc0-ba13-9f9dd2d223b9/”) by Gareth Monger, are both licensed under the Creative Commons Attribution 3.0 Unported license (“http://creativecommons.org/licenses/by/3.0/”).

All of the aligned extension Bionano sequences are aligned to the terminal part of 1p, 3q, 5q, 16q or 19p. We hypothesised that these terminals may themselves share homologous sequences, and searched for regions within subtelomeric regions which have high sub-telomeric copy number. By analysing high copy number repeats in the sequence of subtelomeric regions (see Methods) we identified two sub-telomeric duplication families (family A and B of length 9 kb and 8 kb respectively) which were observed in almost all of the 1p, 3q, 5q, 16q, 19p extension sequence regions. We identified all homologous sequences to these two families in the non-redundant nucleotide database which we used to construct a phylogenetic tree for each family (see Methods, Fig. 3A,B, Tables S6 and S7). These duplications were only found in great apes and were highly duplicated in human (A:16, B:16 copies) and chimpanzee (A:9, B:7 copies). Especially, both families are preferentially duplicated/enriched at subtelomeric regions (p-value <0.005) compared to their distribution in the genome (see Table S9). In human subtelomeric regions, both families are clustered within a branch of 100% bootstrap value. The nucleotide divergence between human subtelomeric copies is 0.021% to 0.79% and 0.013% to 0.58% for family A and B (see Methods), respectively. Assuming nucleotide substitutions occur at a rate of about 1.5% per 10 million years (3% divergence between copies)^20^, families A and B underwent human subtelomeric duplication at 0.069 to 2.64 and 0.042 to 1.95 million years ago which is after the divergence of human and chimpanzee. This is consistent with the observation that both families at chimpanzee subtelomeric regions are clustered with themselves. It suggests that the subtelomeric expansion of families A and B occurred recently and independently in human and chimpanzee lineage, which also supports the hypothesis of chromosome end extension from duplicating subtelomeric regions. Notably, family A recently duplicated not only at the terminal regions but also at interstitial regions (human Y and chimpanzee 6).

A two-step duplication history is inferred from the remaining phylogenetic tree. The first step is interstitial intra-chromosome duplication (4 copies) in ancient chromosome 7 in family B (dark purple in Fig. 3B). The second step is interstitial inter-chromosome duplication from ancient chromosome 7 to another chromosome for both families. Interestingly, these families appeared to both originate from the same region on ancient primate chromosome 7 (chr7:45832681-45863525, 7p12.3), separated only by 13 kb. In family A, the copy at 7p12.3 (indicated by H7i) achieved highest nucleotide divergence of 4.1% (from terminal copies), indicating 7p12.3 was the original copy. In family B, 7p12.3 was not the ancestor copy among another chromosome 7 copy, but the phylogenetic tree (H7i+. B in Fig. 3B) suggested it was the closest related copy to other human inter-chromosome copies (bootstrap value 99.9%), indicating the remaining spread of family B may have originated from this 7p12.3 copy. We aligned to gorilla, orangutan and baboon (see methods) and found that both families were truncated into smaller fragments largely located at ancient chromosome 7. Both families likely originated from ancient rearrangements.

**Figure 4:**
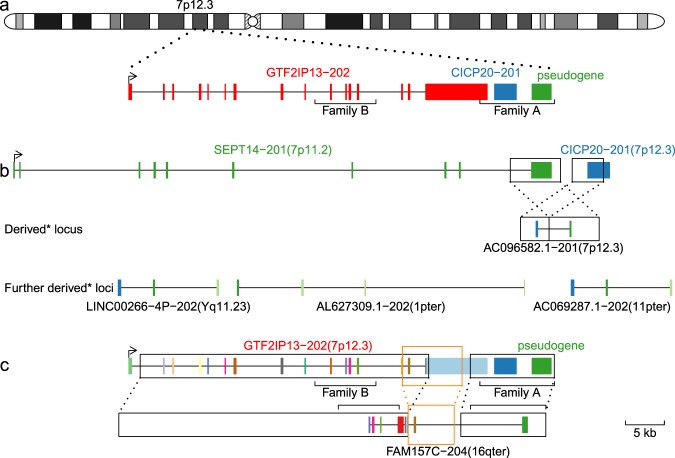
Gene innovation for two sequence families identified in human extension sequences. (**a**) Overview of genes inside ancestral copy. Family A contains last exon of GTF2IP13-202 (red), a pseudogene CICP20-201 (blue) and an unannotated pseudogene (green). Family B contain exons 10-13 of GTF2IP13-202. (**b**) Putative gene fusion in family A. The unannotated pseudogene (green) is homologous to the last exon of SEPT14-201. The AC096582.1-201 is a combination of part of CICP20-201 and part of last exon of SEPT14-201. AC096582.1-201 later combines with sequences from both interstitial and subtelomeric regions into LINC00266-4P-202 (first two exons), AL627309.1-202 (first exon) and AC069287.1-202 (first two exons). Derived* indicates that it is inferred from the phylogentics tree in Fig. 3. (**c**) Gene modification in family B. Each exon of GTF2IP13-202 is colored in different color. Boxes and links are drawn for duplication pairs (inferred from database^20^). FAM157C-204 exons 1-3, 5-6 and 7 are homologous to GTF2IP13-202 exons 11–13, 16-17 and 15. Notably, GTF2IP13-202 exon 15 (brown) is downstream of GTF2IP13-202 exon 16-17 (grey, lightblue) in FAM157C-204. FAM157C-204 exon 4 is homologous to GTF2IP13-202 intron sequence. FAM157C-204 last exon (8) is homologous to SEPT14-201 last exon.

We further investigated the gene annotation of these both families A and B (see Methods). The putative ancestor copy 7p11.12 consists of a 17 exon pseudogene GTF2IP13-202 (Fig. 4a), which is the primary ancestral copy of gene FAM157A, FAM157B and FAM157C (Fig. 3). Family B contains GTF2IP13-202 exons 10–13. Family A contains GTF2IP13-202 exon 17 (last), a pseudogene CICP20 and an unannotated pseudogene. This unannotated pseudogene is homologous to exon 10 (last) of SEPT14-201, possibly derived via an intrachromosome ancient duplication (chr7:45818236-45824692 and chr7:55792353-55798826). As a result, the combination of partial CICP and partial SEPT14-201 becomes AC096582.1-201. The sequence of AC096582.1-201 is the common first or first two exons of multiple transcripts in family A duplication regions, such as LINC00266-4P-202, AL627309.1-202 and AC069287.1-202 (Fig. 4b). The duplication of families A and B also leads to modifications in the ordering of exons. For example, FAM157C-204 exons 1-7 are primarily homologous to the sequence of GTF2IP13-202. But it changes the exon order and caps with the last exon of SEPT14-201 (Fig. 4c). The duplication of families A and B has lead to gene innovation.

## Discussion

In this study, we provide evidence that chromosome ends are dynamic and have undergone substantial extension since the divergence of primates. Our results suggest the process of chromosome-end extension is ongoing and generates significant polymorphism in the population, which is heritable from one generation to the next.

We identified two duplication families which are participating in ongoing chromosome end expansion. These families appear to both originate from the same region on ancient primate chromosome 7, separated only by 13 kb; however, they have undergone distinct patterns of duplication in subtelomeric regions of at least two primates (Chimpanzee and Human). These families are still participating in subtelomeric duplication in human populations, as can be seen by the alignment of these families to observed BioNano chromosome extension sequence. We showed that duplication of draftregions containing these families in sub-telomeric regions has led to the formation of new fusion genes. This may provide a mechanism for the extension sequence to be adaptively selected and eventually become fixed in the population.

Chromosome end diploid assembly is extremely challenging. For example, NA12878 15q (Fig. 1b) extension sequence can be inferred to be heterozygous in the sample based on the observation of the extension sequence in its parents. This could also be validated from the presence of both reference-supporting and extension-supporting raw BioNano read alignments (11 for reference and 16 for extension). The IrysSolve assembler assumes the non-extended reference type is an incomplete sequence and assembles the contig to the longest extended one. The shorter sequence is therefore ignored. The diploid sequence will likely be chopped into small pieces. If the sequence is not complex and long enough, it will output as contig, such as 4q (Figure S3). Otherwise, it is hard to track the diploid sequence. This is the difficulty to determine the zygosity of every chromosome end.

We noticed there is a recent paper about chromosome terminus^21^, which focuses on the telomere length estimation using Bionano data. Both Young et.al and this manuscript report polymorphism of chromosome ends, such as 15q extension at NA12878 and NA12892 and diploid 4q sequence. Our manuscript further realigns these extension sequences (regarded as haplotypes, gaps or structural variations in^21^) from BioNano labels and also validates extension sequence using nanopore sequence data. We also identify genomic regions within extension sequence which have been subject to ongoing duplication via sub-telomeric extension throughout primate evolution.

We have uncovered extensive subtelomeric sequence variation from a quite limited sample consisting of 3 trios and 2 unrelated samples. It is likely that further study of chromosome terminal regions from larger sample sizes, using either BioNano optical mapping or long-read sequencing will uncover substantially more subtelomeric sequence variation.

## Methods

### Bionano data analysis

We downloaded publicly available BioNano data for eight humans (a CEPH trio, an Ashkenazi trio and two Han Chinese) from BioNano Genomics website http://bionanogenomics.com/science/public-datasets/. The initial downloaded data contained raw Bionano data, assembled contigs as well as the unique alignments from contigs to GRCh37 generated using RefAlign from BioNano Genomics IrysSolve. The raw BioNano read depth (3.1G as a fold) are 91X(NA12878), 76X(NA12891), 70X(NA12892), 100X(NA24143), 90X(NA24149), 120X(NA24385), 58X(NA24631) and 72X(YH). The assembler assembles reads by the overlap-layout-consensus pattern. The reads overlapping score is quantified by a maximum likelihood model. Optical maps are aligned by a dynamic programming approach. The scoring function of this approach is the likelihood of a pair of label intervals being similar (for details see^22^).

This alignment mapped each Bionano contig to the best matching position on GRCh37 and did not require a full-length alignment of the contig to reference, thus allowing us to identify chromosome end polymorphism. We assumed that contigs which are aligned to the most distal sequence with the highest matching score represent the individual sample chromosome ends. As the technology is currently unable to reliably resolve different chromosome end haplotypes, the chromosome ends can be viewed as a dominant marker for the longest allele.

The reference chromosome end sequences which could not be aligned by are defined as missing sequences. The reference unknown sequences (“N” region) is removed for missing region size estimation. The unaligned sequence at the distal part of the individual chromosome end is defined as extension sequence. We imposed a length threshold of 33.1 kb (average labels distance + 3*standard deviation) in order to define either missing or extension sequence, otherwise, the chromosome end was considered reference-type. Chromosomes 13p, 14p, 15p, 21p, 22p were removed because of missing reference sequence. 1p is removed when all the sample’s contigs are discontinuous at the reference unknown region (chr1:471k-521k) and the remaining of 1p could only be aligned as secondary alignment. Sex chromosomes are regarded as one chromosome due to the extensive homology of chromosome ends.

In order to recognize the origin of the extension sequences, we realigned the contigs to GRCh37 (373, 590 labels) allowing multiple matches by RefAlign. A pre-alignment process in RefAlign was used to merge labels which were close (450 bp) to each other. This process resulted in the identification of 346, 991 labels for subsequent analysis. The mean distance and standard deviation between adjacent labels was 8.2 kb and 8.3 kb, respectively. The adjacent labels which contain reference gap regions are excluded in estimation. We then used RefAlign to re-align the contigs to the GRCh37 (without chromosome Y) reference enzyme recognition sites (RefAlign parameter: -output-veto-filter _intervals.txt$ -res 2.9 -FP 0.6 -FN 0.06 -sf 0.20 -sd 0.0 -sr 0.01 -extend 1 -outlier 0.0001 -endoutlier 0.001 -PVendoutlier -deltaX 12 -deltaY 12 -hashgen 5 7 2.4 1.5 0.05 5.0 1 1 1 -hash -hashdelta 50 -mres 1e-3 -hashMultiMatch 100 -insertThreads 4 -nosplit 2 -biaswt 0 -T 1e-12 -S -1000 -indel -PVres 2 -rres 0.9 -MaxSE 0.5 -HSDrange 1.0 -outlierBC -AlignRes 2. -outlierExtend 12 24 -Kmax 12 -f -maxmem 128 -BestRef 0 -MultiMatches 5). This re-alignment allowed us to more accurately identify chromosome ends. In particular, for 6p in sample NA12878, the multiple alignments supported a more distal alignment (chr6:73k-364k) than the unique alignment (chr6:245k-1870k). The break of these two contigs was mediated by a connection from 6p to a chromosome 16 interstitial region, which could be an artefact. Similarly, for 16p in NA24385, the multiple alignments supported a more distal alignment (chr16:67k-204k) than the unique alignment (chr16:204k-3728k). The more distal contig could align to both 19p and 16p with no overlap, but the alignment at 19p (chr19: 61k-244k) is longer than 16p making it primarily align to 19p in unique alignment. Notably, the contig with the highest matching score for 19p in this sample is another contig (chr19:61k-2341k).

We also realigned individual contigs to other contigs with the same parameter as above. When a label in one sample was aligned to another sample label, we regarded these two labels as connected. For duplication family A and B, we extracted the label IDs from GRCh37 label map. Family A contained 3 to 4 labels. Family B contained 2 labels. For the alignment regions containing family A or B, we only drew these regions when at least 3 or 2 labels were aligned to GRCh37, respectively.

For the CEPH and Ashkenazi trios, we checked the alignments from the child to their parent for every chromosome end (Figure S1). If the child chromosome end was aligned to one of its parent with no more than one label difference at the terminus, we considered that the child chromosome end was inherited from the parent.

For every contig, we consider the location of the highest alignment score as the true location of this sequence. We then check whether any termini receive more than one contig. The terminus of 4q in two trios offspring contain two distinguishable sequences (see Figure S3).

### Extension validation from nanopore data

Nanopore data from individual NA12878 (rel4) was download from https://github.com/nanopore-wgs-consortium/NA12878. We aligned these data to GRCh37 by minimap2 (https://github.com/lh3/minimap2) using default parameters. The rel4 raw data contains 21 billion bp (about 7 fold coverage) from 9.4 million reads. See Figure S6 for the read length distribution. 47k reads (5%) aligned more than 100 kb in the reference. The longest aligned read is 939497 bp. We search for the most distal alignments at the terminus and identify the terminal supporting reads. Then we search whether the extended part of these terminal supporting reads align to the known reference sequence. This analysis enables us to determine the unknown sequence in 15q and 20q as tandem duplication of its terminus and 9p, respectively. The alignment details are shown in Table S10.

Although the BioNano data and nanopore data could not be directly aligned to each other, we could predict the sequence and use it to connect the two technologies. If the extension sequence is aligned in the BioNano assembly, it is predicted as a connection of the aligned sequence and terminal sequence with an estimated gap (“N”). If not, we used the aligned nanopore sequence (15q and 20q) instead if applicable. Otherwise, this extension sequence (4q) is unable to compare and validate. These extension termini are listed in Table S5 and available for download at https://github.com/haojingshao/Extension. Then we calculate the BioNano labels in the predicted extension sequence by software in IrysSolve and aligned them to its assembly (as above, seen as a grey link in Fig. 2). The dot plot was generated from the last^23^ (default) alignment of nanopore reads (y-axis) against predicted extension sequence (x-axis). The validation is the correct alignment of both technologies to the same sequence.

### Subtelomeric duplication analysis

We downloaded all pairs of regions in the human genome (GRCh37) with high sequence identity from the segmental duplication database^20^. We assign the base-pair copy number of each position to be the number of entries in this file which overlap this position. We take all positions with base-pair copy number at least 22 to be high copy number regions. We order these regions by their base-pair length, defined as the longest contiguous stretch of DNA with base-pair level copy number greater than 22. We then consider only those duplication copies which span the entire region as members of the family. Only duplication families which have at least one member within 1 MB of a chromosome end, were kept (Table S8). This resulted in an identification of two duplication families with high copy number, which was selected for evolutionary analysis.

We used NCBI blast to align these two sequences (GRCh37, A:chr1:652579-662291, B:chr16:90220392-90228245) to the human genome, chimpanzee genome and nucleotide collection (NT) database, filtering out sequences with less than 95% (human and chimpanzee) or 85% (other) sequence identity to the full query sequence. Next, we extracted the duplications, aligned them by mafft^24^ [key option: strategy G-INS-i]. Then we built the maximum likelihood tree by MEGA7^25^ with 1000 bootstrap iterations. Finally, the tree was drawn by the online version of TreeDyn^26^. These duplication sequences were also aligned to themselves by lastz^27^ (version 1.04.00, parameter default). Then the nucleotide divergence was calculated from the aligned pair (size >9000 and 7000 for family A and B). Indels were ignored in the estimation. Notably, nucleotide collection (NT) database only contains the sequence of GRCh38, thus we report them in GRCh38 coordinate in this section.

Subtelomeric duplication regions are defined as the longest contiguous duplication sequence starting from the chromosome terminus. From the duplication database^20^, both pairs of duplications regions inside subtelomeric duplication regions are defined as subtelomeric duplications. The longest autosome subtelomeric duplication pairs are chr1:317720-471368 and chr5:180744335-180899425. Homology between the X and Y chromosomes in p arm terminus is maintained by an obligatory recombination in male meiosis^28^. This results in about 2.6 Mb almost 100% identity homologous sequence. Almost all of these sequences are only homologous between X and Y. Since the pattern and mechanism are different between autosome and sex chromosome subtelomeric duplication, sex chromosome is excluded from this analysis.

For all duplication within families A and B, we use lastz^23^ (version:v1.04.00, default) to align each duplication to the remaining duplications in the family. Divergence is calculated as the percentage of single base pair difference between two duplications. Insertion and deletion are ignored. Duplication evolutionary history is inferred as the divergence divided by duplication nucleotide substitutions rate (3% per million between copies).

We downloaded the annotation file from GENCODE (GRCh38, v27)^29^. We used lastz (default parameter)^27^ to align exons sequences to themselves. We regarded exon A was homologous to exon B when 90% sequences of exon A was aligned to exon B with identity more than 95%.

## Electronic supplementary material

Supplementary Figures

Supplementary Table
