# Supplementary material for: Ongoing human chromosome end extension revealed by analysis of BioNano and nanopore data
Source: Sci Rep. 2018 Nov 9;8:16616. doi: 10.1038/s41598-018-34774-0 (PMC6226469; doi:10.1038/s41598-018-34774-0)
Supplement: Supplementary file 1 — Supplementary Figures [file 41598_2018_34774_MOESM1_ESM.pdf]

Ongoing human chromosome end extension revealed by analysis of BioNano and nanopore data

Haojing Shao[1], Chenxi Zhou[1], Minh Duc Cao[1], and Lachlan J.M. Coin[1,*]

[1]Institute for Molecular Bioscience, University of Queensland, St Lucia, Brisbane, QLD 4072 Australia
[*]Correspondence: l.coin@imb.uq.edu.au

List of Figures

S1 **Paralogy map for BioNano assembly chromosome terminal regions in eight samples.** 2-9
S2 **Homology map and label map for 2q and 9q.** 10
S3 **Chromosome 4q diploid sequence in BioNano assembly from two trios.** 11
S4 **Comparison of two terminals from bionano assembly and nanopore reads.** 12
S5 **Self dotplots for five nanopore reads.** 13
S6 **Histogram of raw nanopore read length.** 14

1

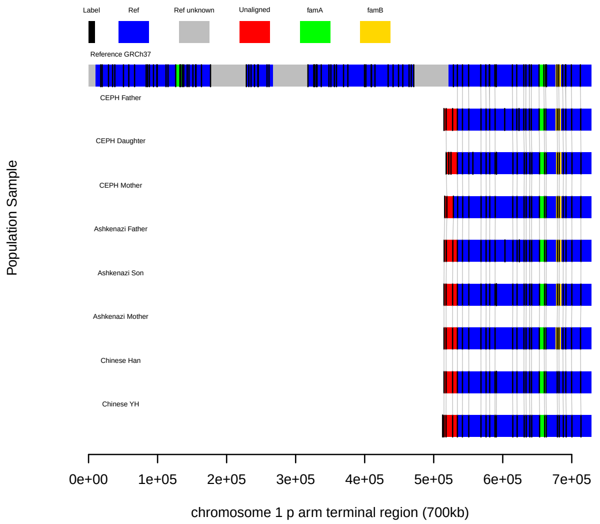
Label
Ref
Ref unknown
Unaligned
famA
famB
Reference GRCh37
CEPH Father
CEPH Daughter
CEPH Mother
Ashkenazi Father
Ashkenazi Son
Ashkenazi Mother
Chinese Han
Chinese YH
Population Sample
0e+00
1e+05
2e+05
3e+05
4e+05
5e+05
6e+05
7e+05
chromosome 1 p arm terminal region (700kb)

2

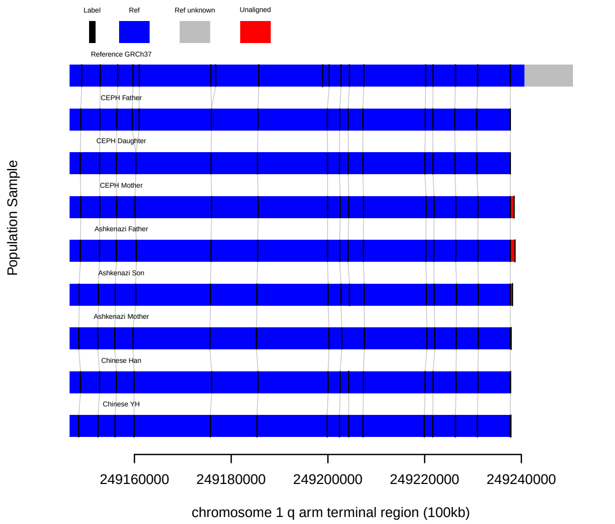
Label
Ref
Ref unknown
Unaligned
Reference GRCh37
CEPH Father
CEPH Daughter
CEPH Mother
Ashkenazi Father
Ashkenazi Son
Ashkenazi Mother
Chinese Han
Chinese YH
Population Sample
249160000
249180000
249200000
249220000
249240000
chromosome 1 q arm terminal region (100kb)

3

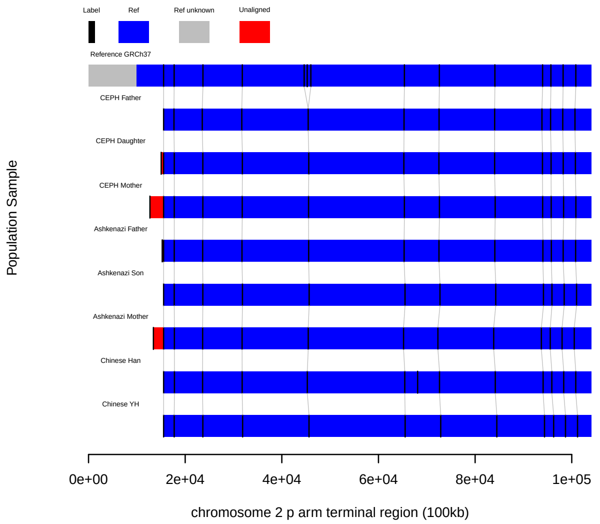
Label
Ref
Ref unknown
Unaligned
Reference GRCh37
CEPH Father
CEPH Daughter
CEPH Mother
Ashkenazi Father
Ashkenazi Son
Ashkenazi Mother
Chinese Han
Chinese YH
Population Sample
0e+00
2e+04
4e+04
6e+04
8e+04
1e+05
chromosome 2 p arm terminal region (100kb)

4

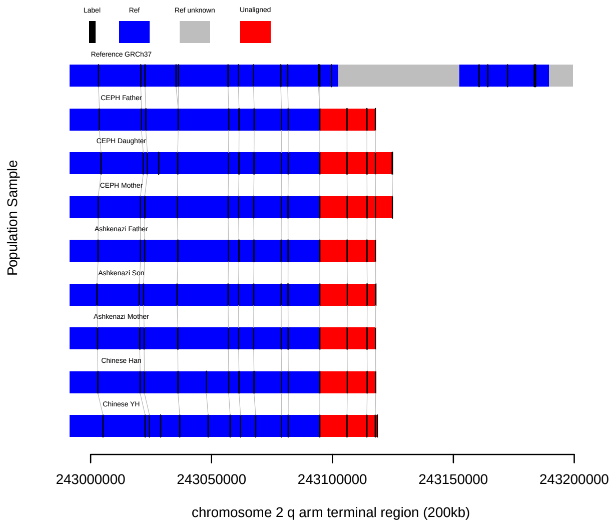
Label
Ref
Ref unknown
Unaligned
Reference GRCh37
CEPH Father
CEPH Daughter
CEPH Mother
Ashkenazi Father
Ashkenazi Son
Ashkenazi Mother
Chinese Han
Chinese YH
Population Sample
243000000
243050000
243100000
243150000
243200000
chromosome 2 q arm terminal region (200kb)

5

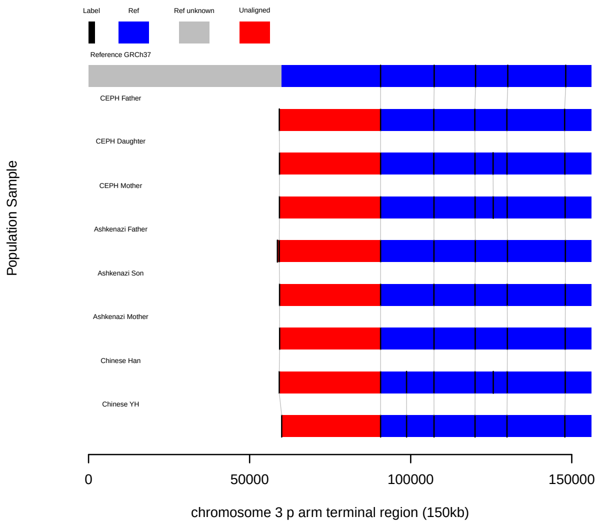
Label
Ref
Ref unknown
Unaligned
Reference GRCh37
CEPH Father
CEPH Daughter
CEPH Mother
Ashkenazi Father
Ashkenazi Son
Ashkenazi Mother
Chinese Han
Chinese YH
Population Sample
0
50000
100000
150000
chromosome 3 p arm terminal region (150kb)

6

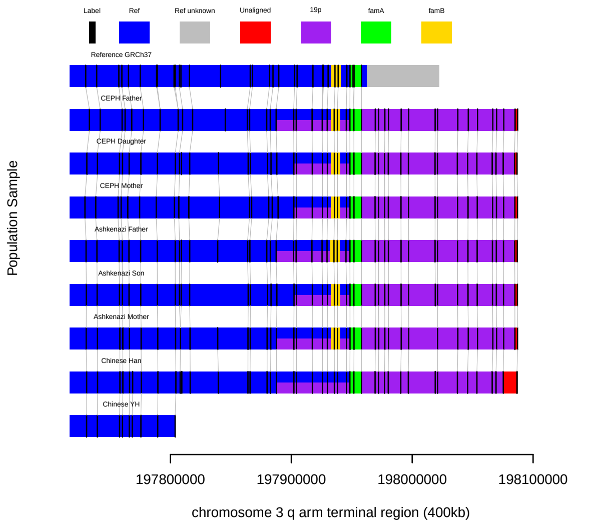
Label
Ref
Ref unknown
Unaligned
19p
famA
famB
Reference GRCh37
CEPH Father
CEPH Daughter
CEPH Mother
Ashkenazi Father
Ashkenazi Son
Ashkenazi Mother
Chinese Han
Chinese YH
Population Sample
197800000
197900000
198000000
198100000
chromosome 3 q arm terminal region (400kb)

7

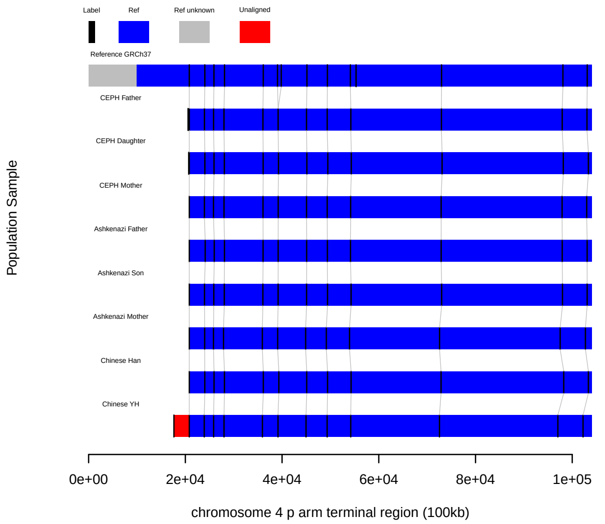
Label
Ref
Ref unknown
Unaligned
Reference GRCh37
CEPH Father
CEPH Daughter
CEPH Mother
Ashkenazi Father
Ashkenazi Son
Ashkenazi Mother
Chinese Han
Chinese YH
Population Sample
0e+00
2e+04
4e+04
6e+04
8e+04
1e+05
chromosome 4 p arm terminal region (100kb)

8

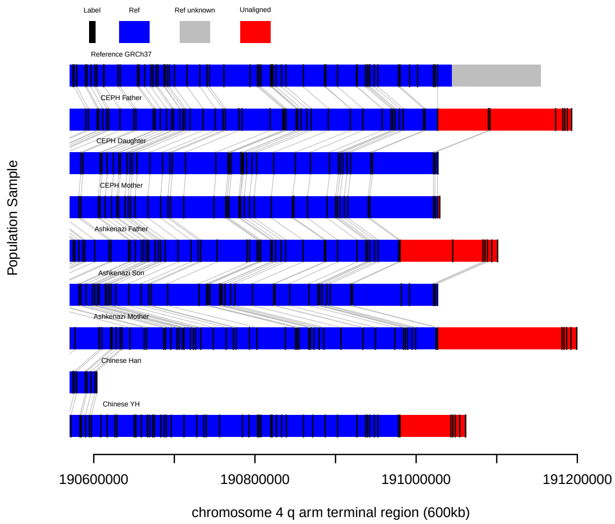
Label
Ref
Ref unknown
Unaligned
Reference GRCh37
CEPH Father
CEPH Daughter
CEPH Mother
Ashkenazi Father
Ashkenazi Son
Ashkenazi Mother
Chinese Han
Chinese YH
Population Sample
190600000
190800000
191000000
191200000
chromosome 4 q arm terminal region (600kb)

9

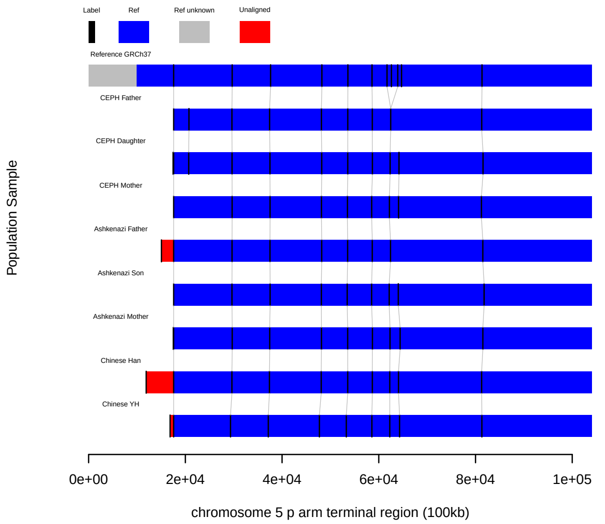
Label
Ref
Ref unknown
Unaligned
Reference GRCh37
CEPH Father
CEPH Daughter
CEPH Mother
Ashkenazi Father
Ashkenazi Son
Ashkenazi Mother
Chinese Han
Chinese YH
Population Sample
0e+00
2e+04
4e+04
6e+04
8e+04
1e+05
chromosome 5 p arm terminal region (100kb)

10

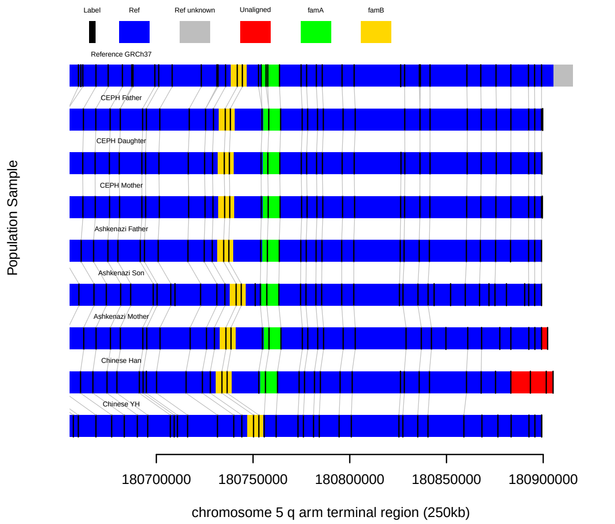
Label
Ref
Ref unknown
Unaligned
famA
famB
Reference GRCh37
CEPH Father
CEPH Daughter
CEPH Mother
Ashkenazi Father
Ashkenazi Son
Ashkenazi Mother
Chinese Han
Chinese YH
Population Sample
180700000
180750000
180800000
180850000
180900000
chromosome 5 q arm terminal region (250kb)

11

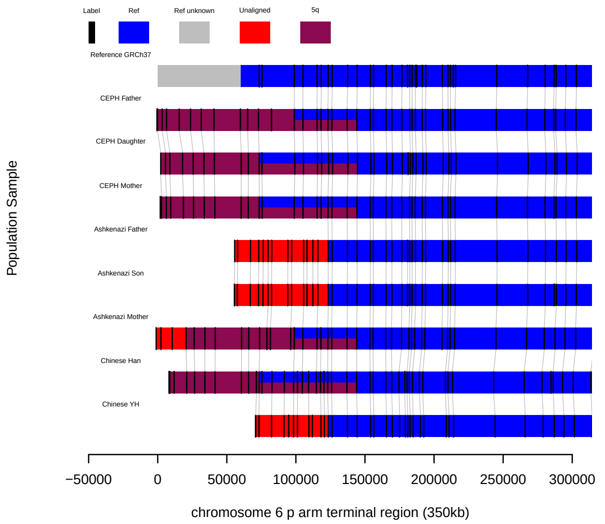
Label
Ref
Ref unknown
Unaligned
5q
Reference GRCh37
CEPH Father
CEPH Daughter
CEPH Mother
Ashkenazi Father
Ashkenazi Son
Ashkenazi Mother
Chinese Han
Chinese YH
Population Sample
−50000
0
50000
100000
150000
200000
250000
300000
chromosome 6 p arm terminal region (350kb)

12

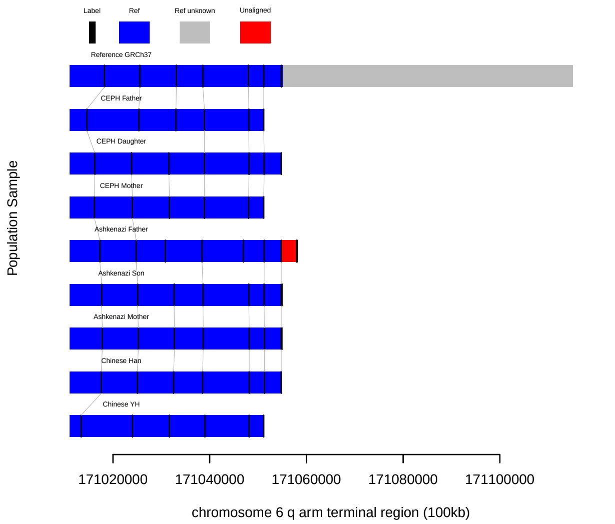
Label
Ref
Ref unknown
Unaligned
Reference GRCh37
CEPH Father
CEPH Daughter
CEPH Mother
Ashkenazi Father
Ashkenazi Son
Ashkenazi Mother
Chinese Han
Chinese YH
Population Sample
171020000
171040000
171060000
171080000
171100000
chromosome 6 q arm terminal region (100kb)

13

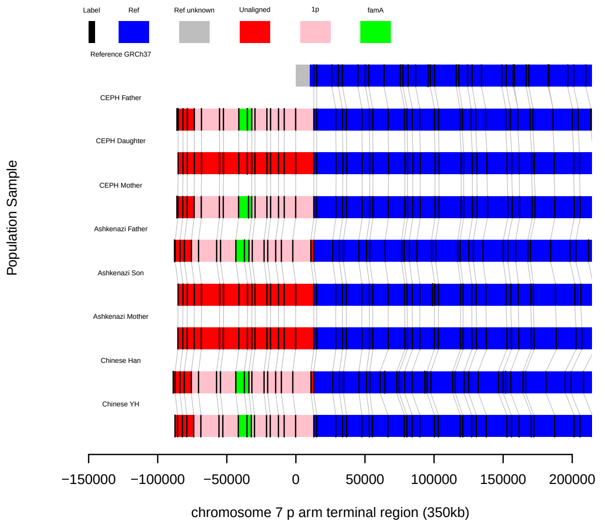

14

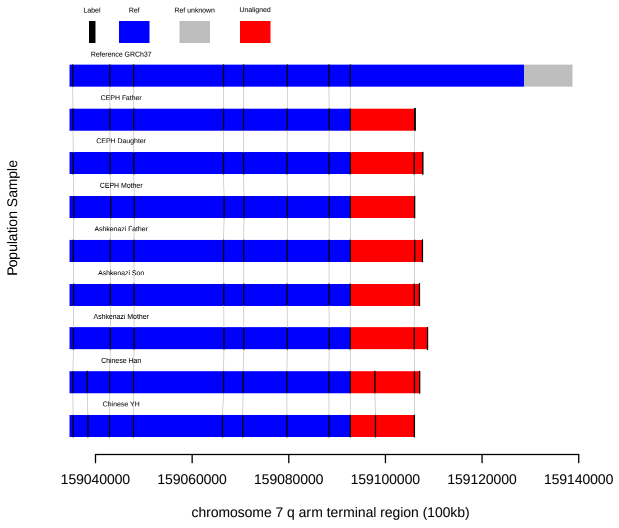

15

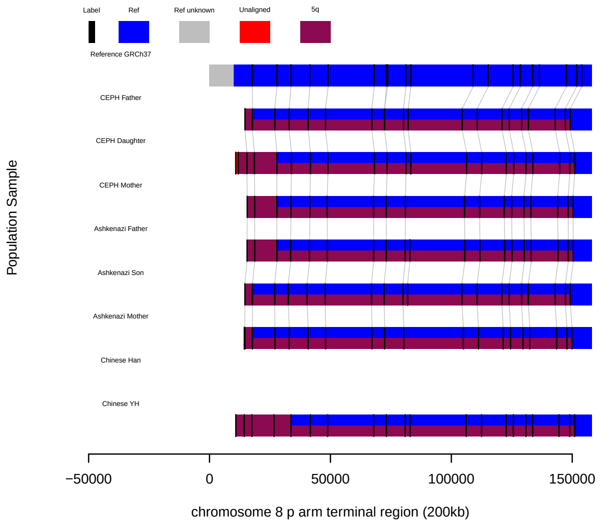

16

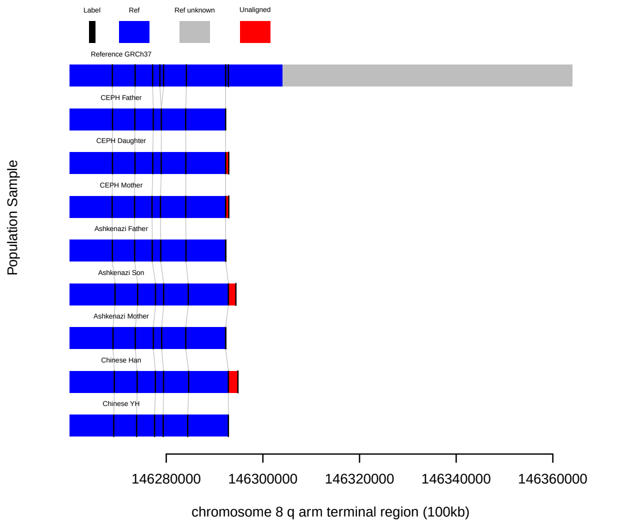

17

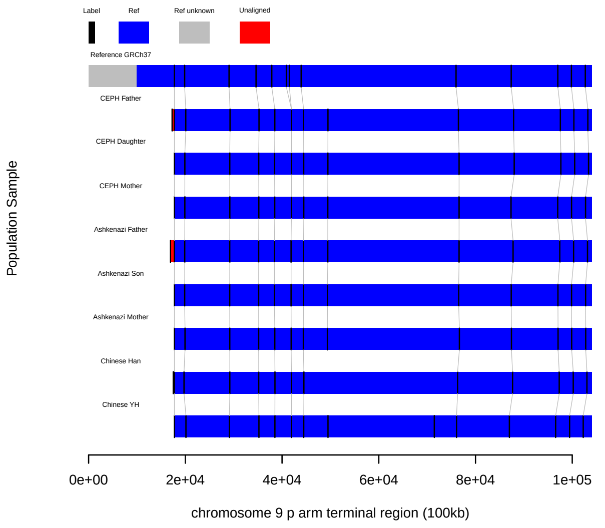

18

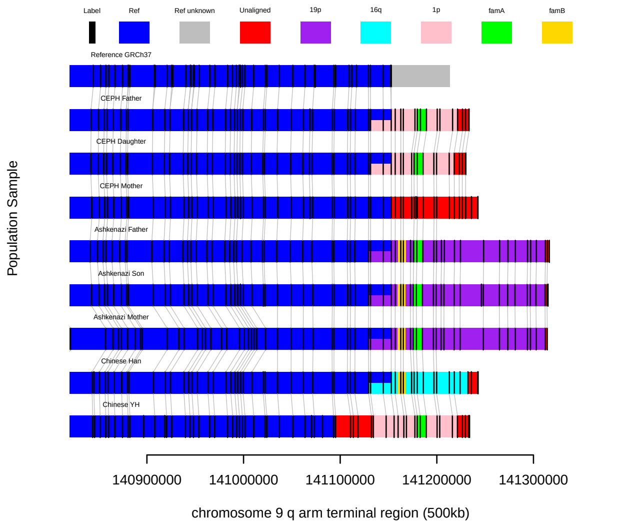

19

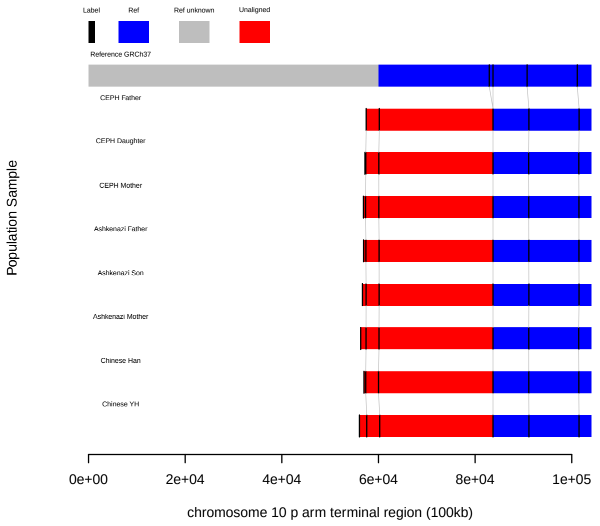

20

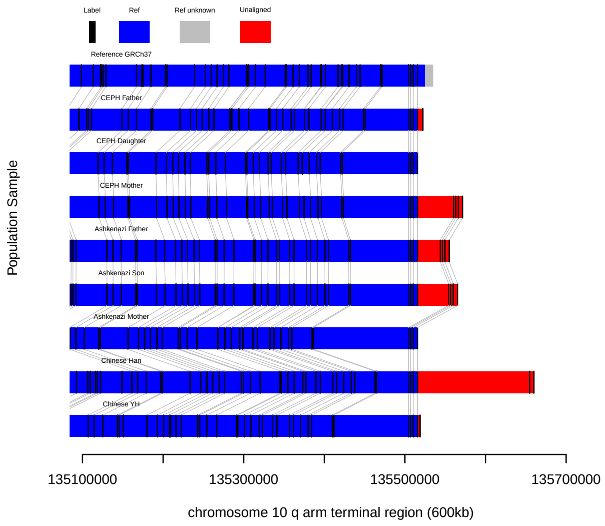

21

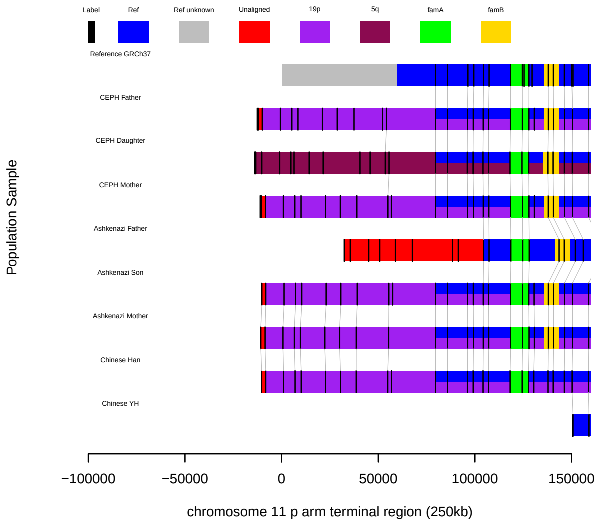

22

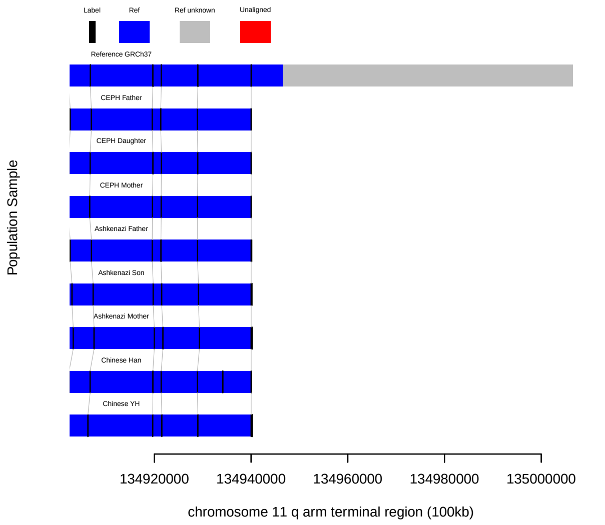

23

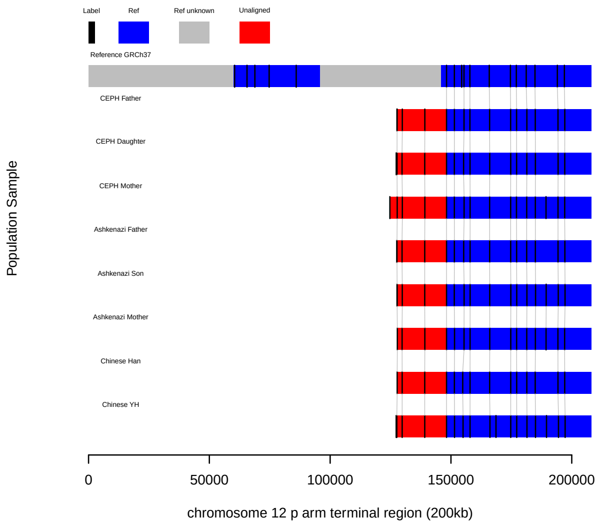

24

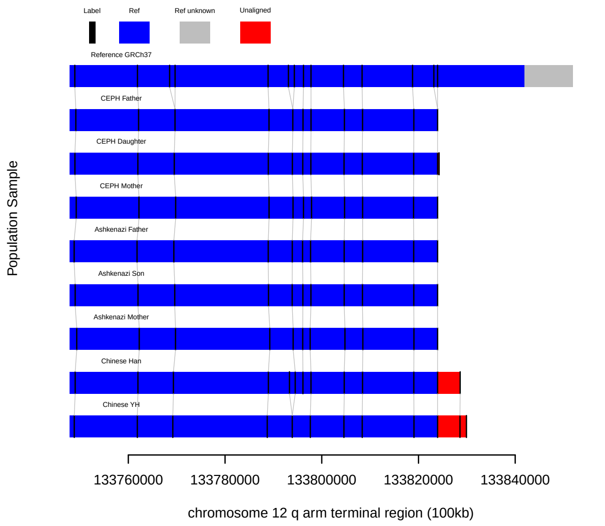

25

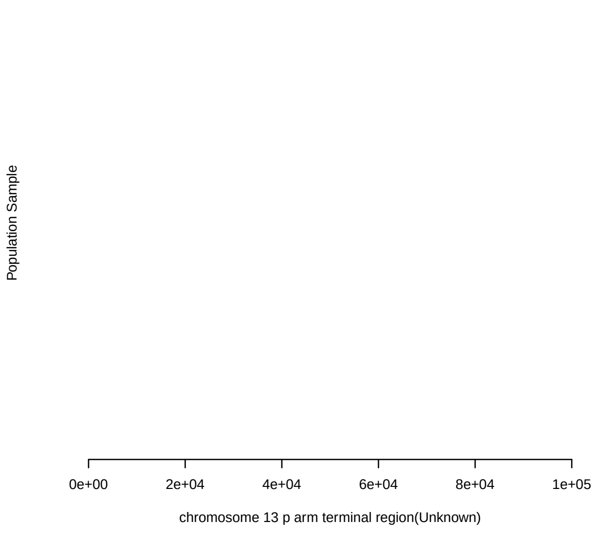

26

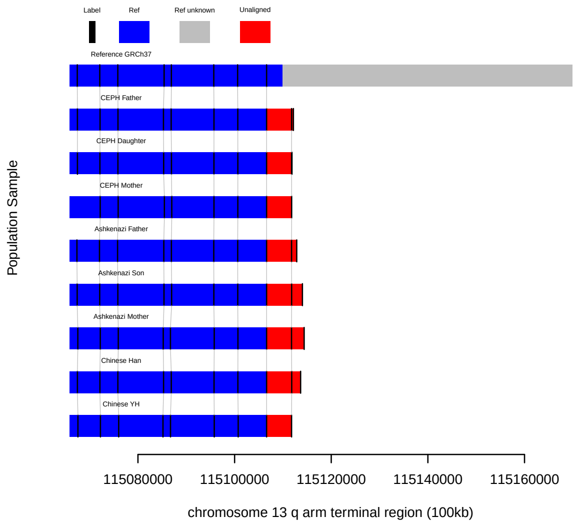

27

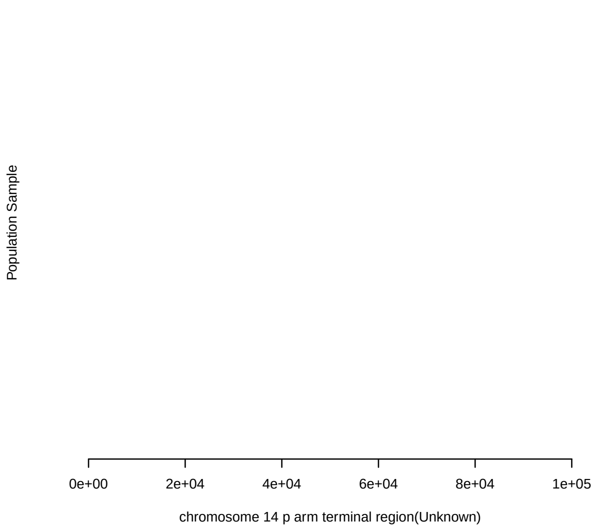

28

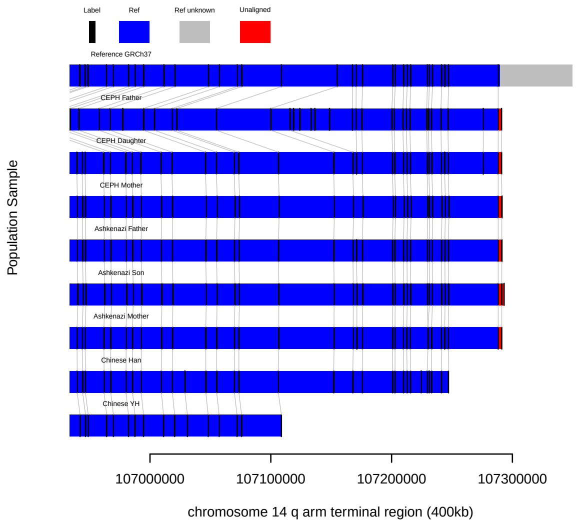

29

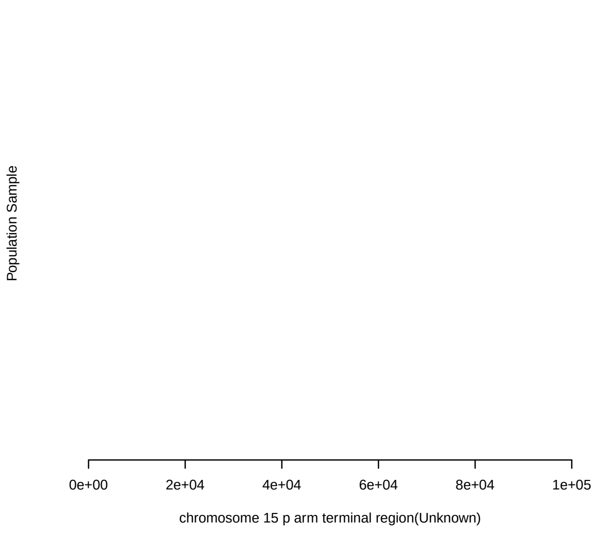

30

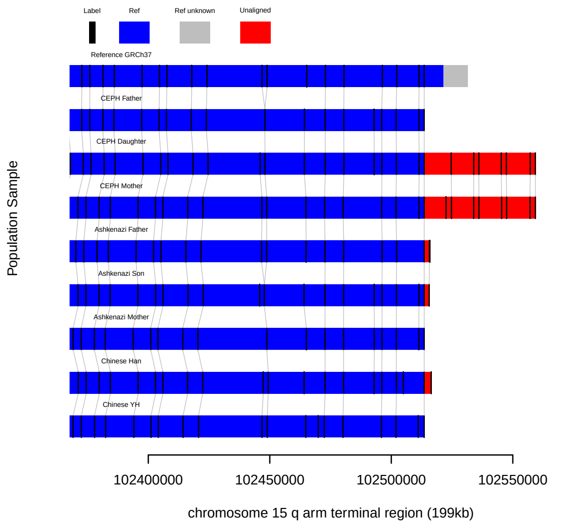

31

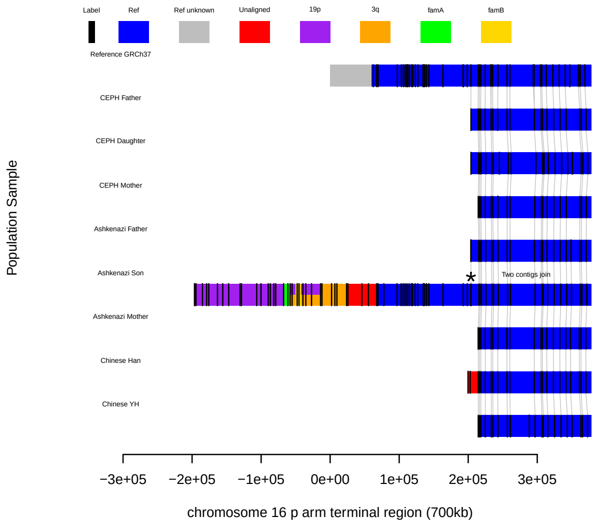

32

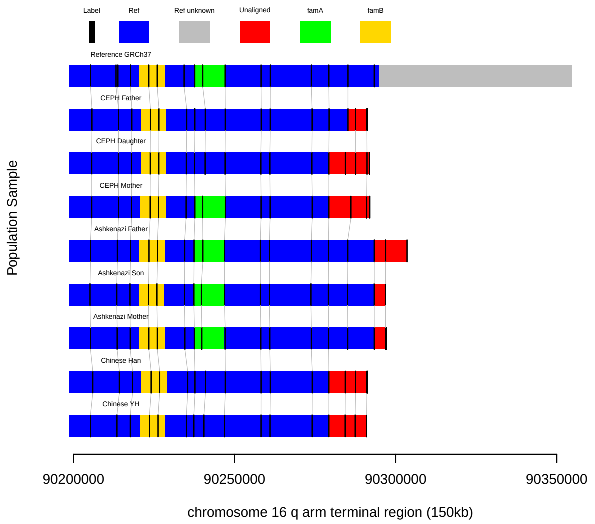

33

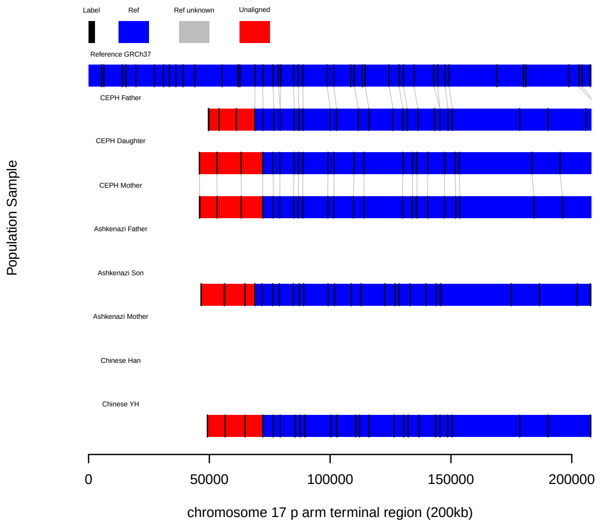

34

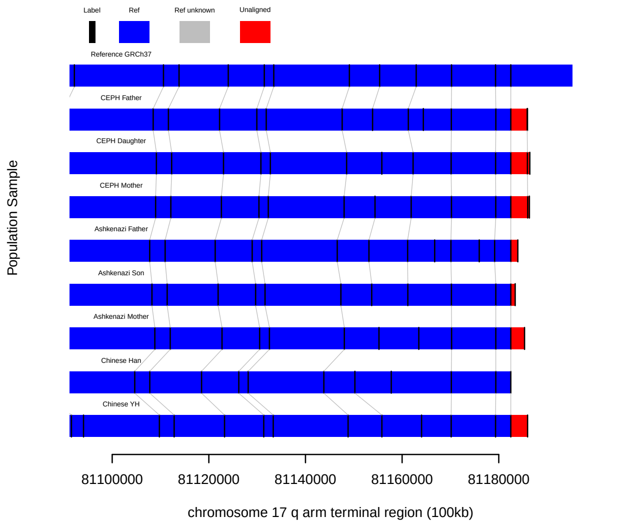

35

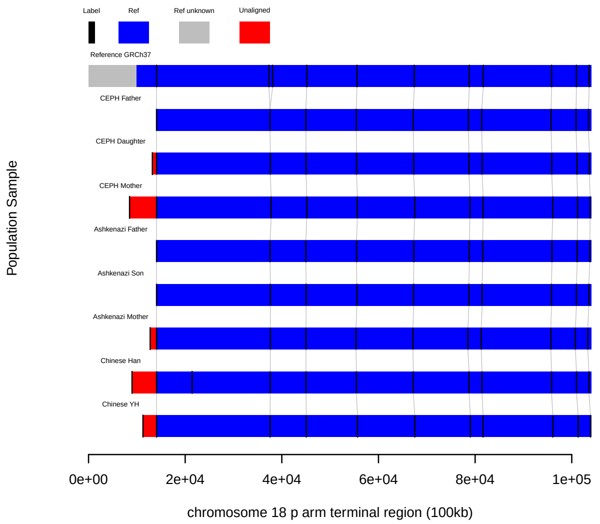

36

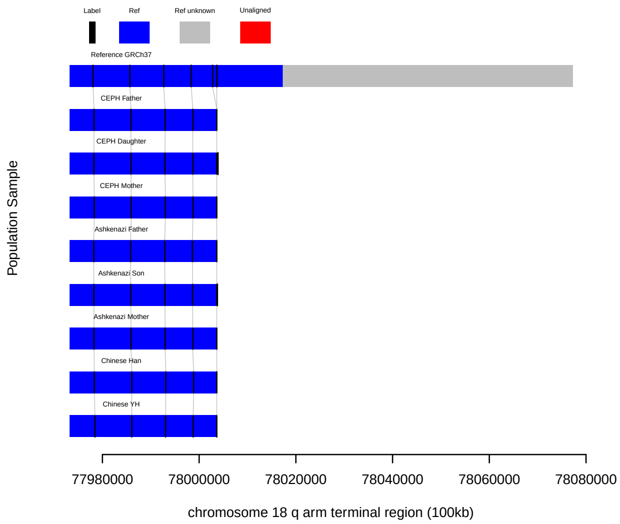

37

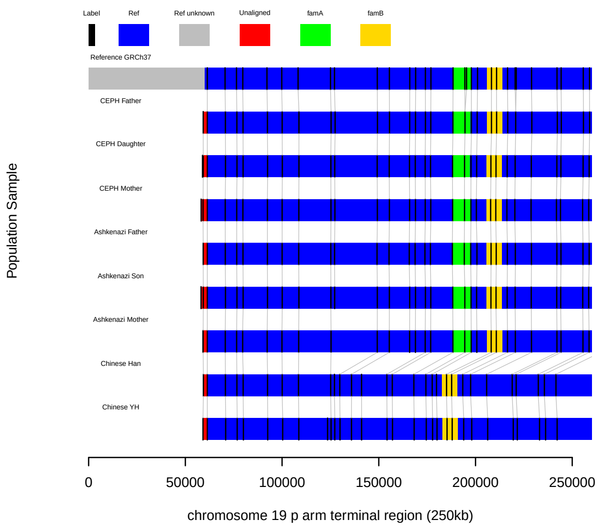

38

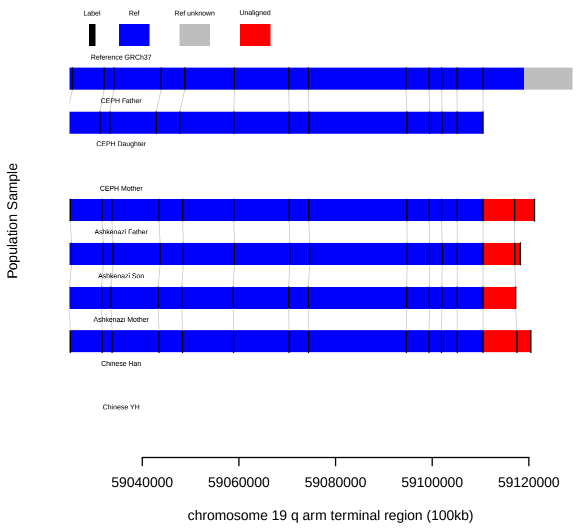

39

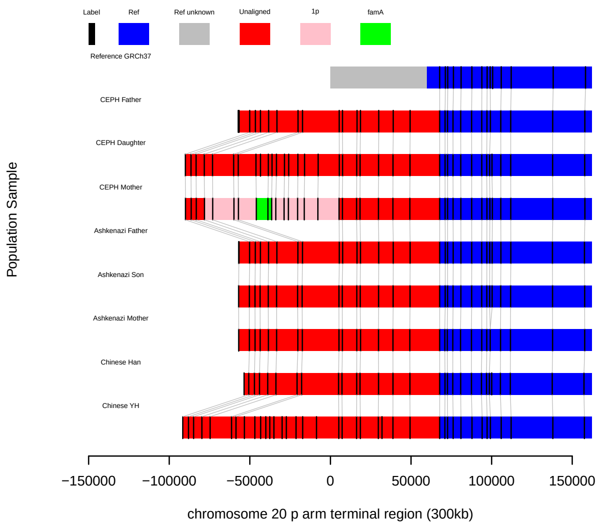

40

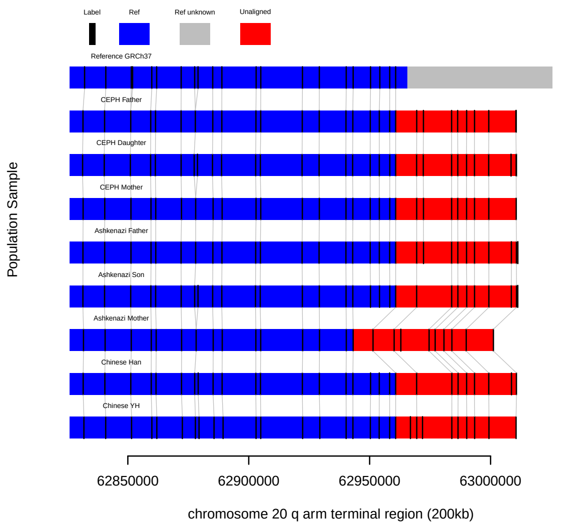

41

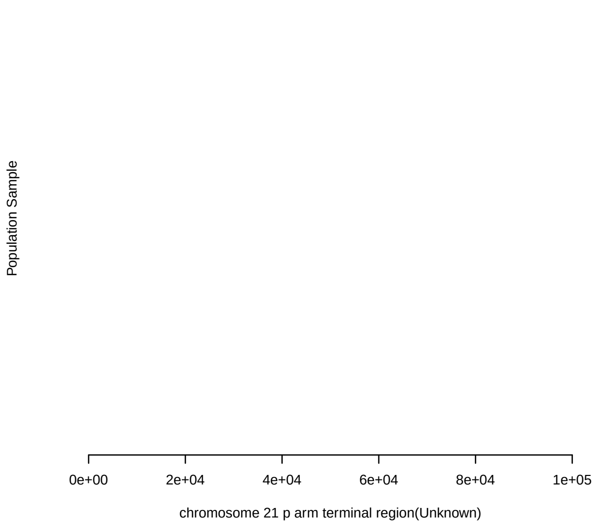

42

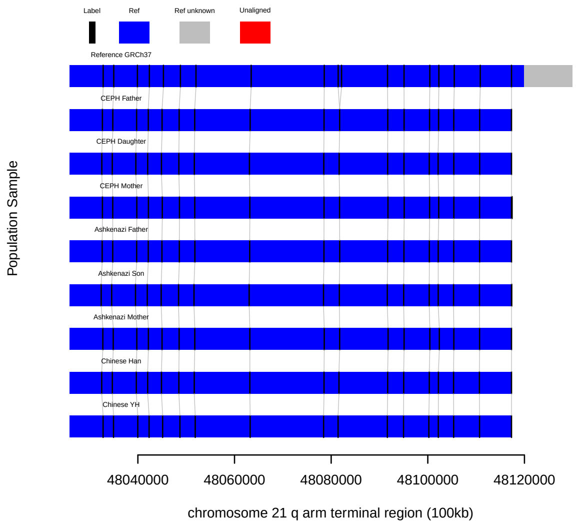

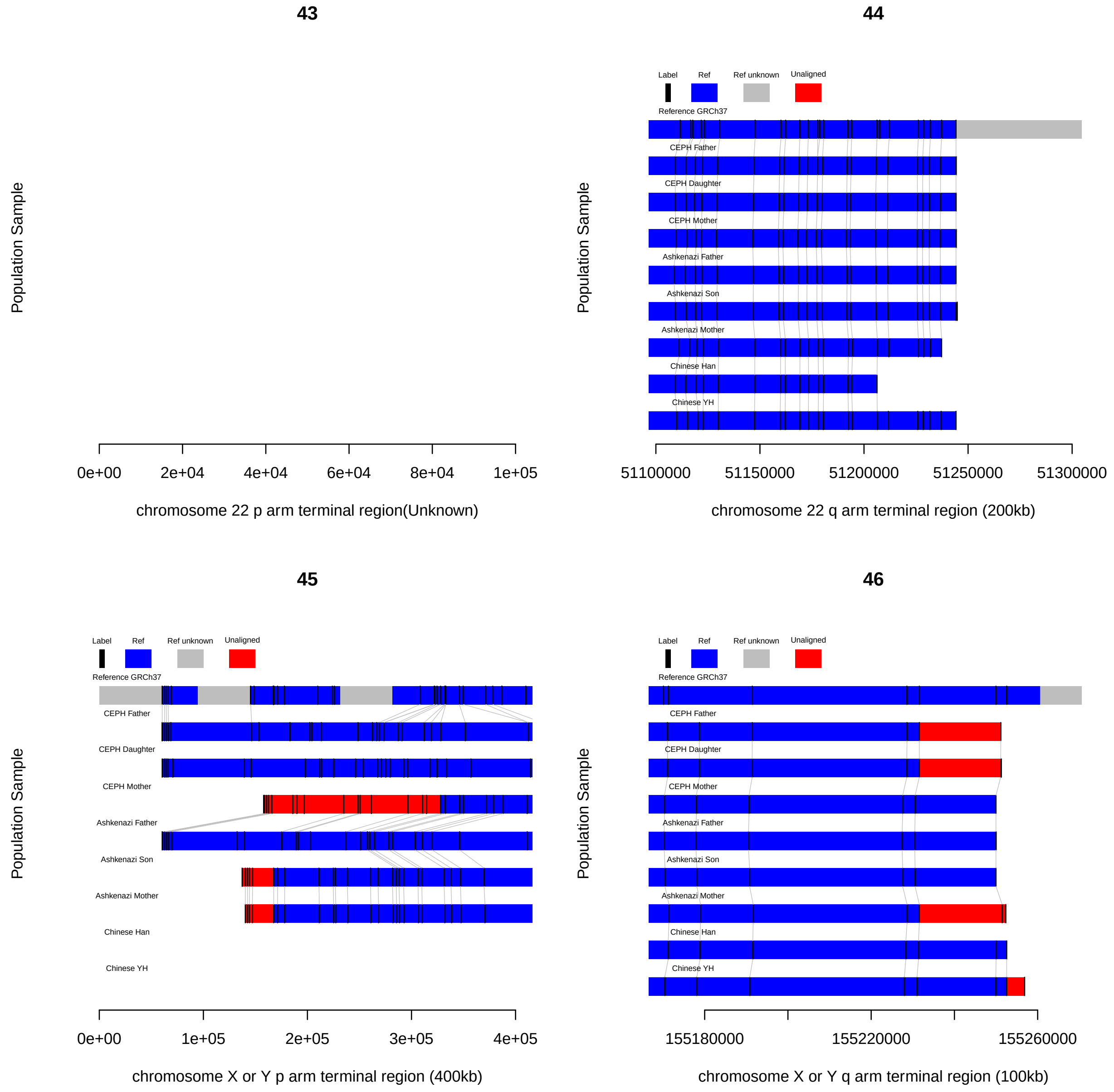

Figure S1. Paralogy map for BioNano assembly chromosome terminal regions in eight samples. *Sub figure 1 to 46 are for 46 human chromosome terminal region. The terminal region of chromosome X and Y are merged into one. Each contig is drawn as a color block, where the color indicates homology, and overlapping colors indicate homology to two sources. The enzyme recognition sites (labels) are marked as black bars. The homology sequence is drawn with the same color. Blue is for human reference or its homology, grey for reference unknown sequence, purple for homology to 19p(chr19:61k-244k), cyan for homology to 16q(chr16:90190k-90293k), pink for homology to 1p(chr1:94k-177k), deep pink for homology to 5q(chr5:180708k-180899k, homologous to chr1:327k-471k and chr1:576k-712k). The remaining unaligned regions are all colored with red. The gray line connections between labels indicate they are aligned to each other(see Methods). The unknown chromosome tip regions are drawn as blank. Five extension sequence contigs (CEPH daughter: 8p,11p,19q and Ashkenazi son:16p,17p) are not inherited.*

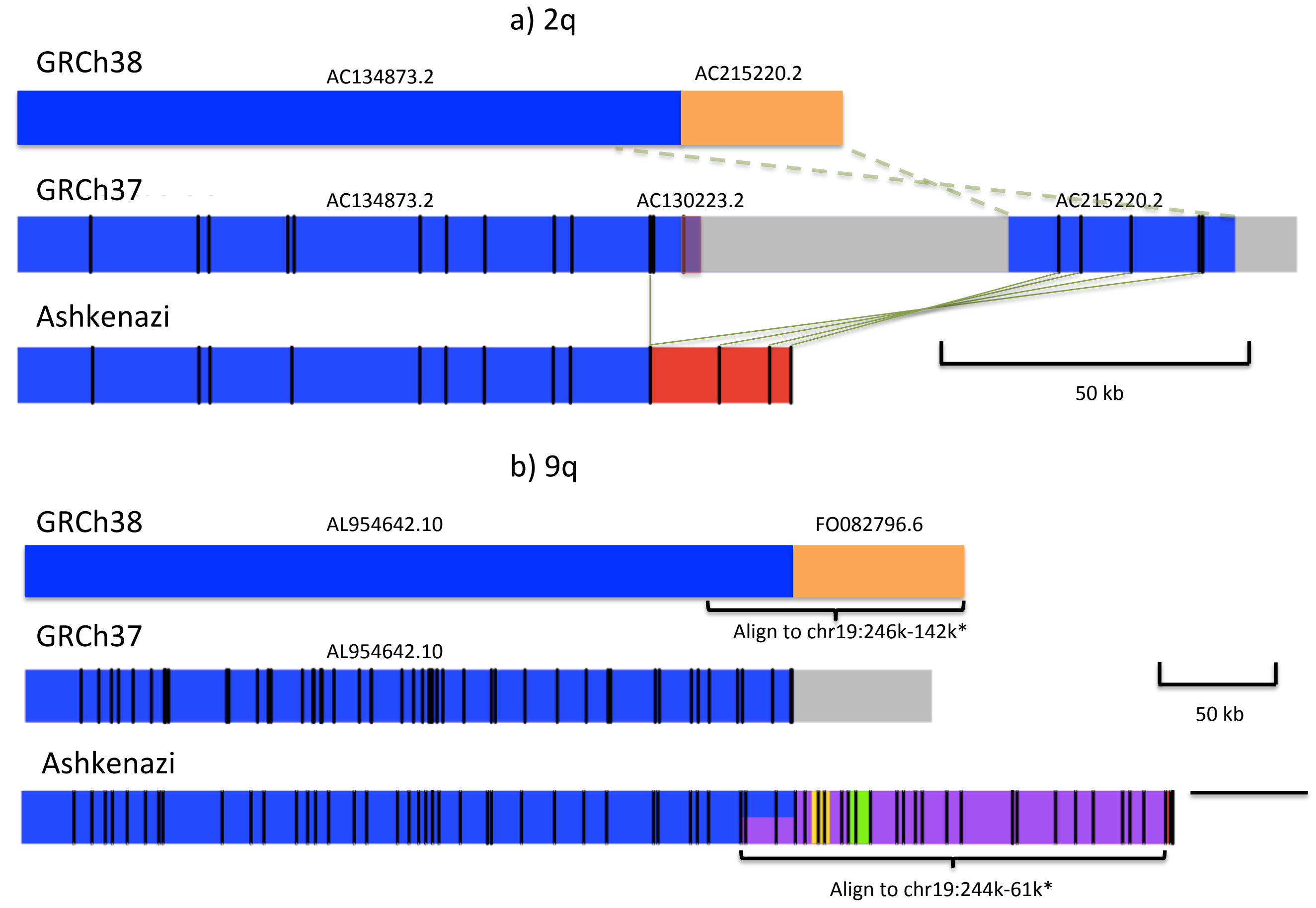

Figure S2. Homology map and label map for 2q and 9q.

*Scaffolds ID are on the top of the sequence. Scaffold AC130223.2 is colored in purple. Dash green lines indicate homologous sequence. Solid light green line indicate the labels are aligned. * means reverse alignment. Labels are obtained from Figure 1.*

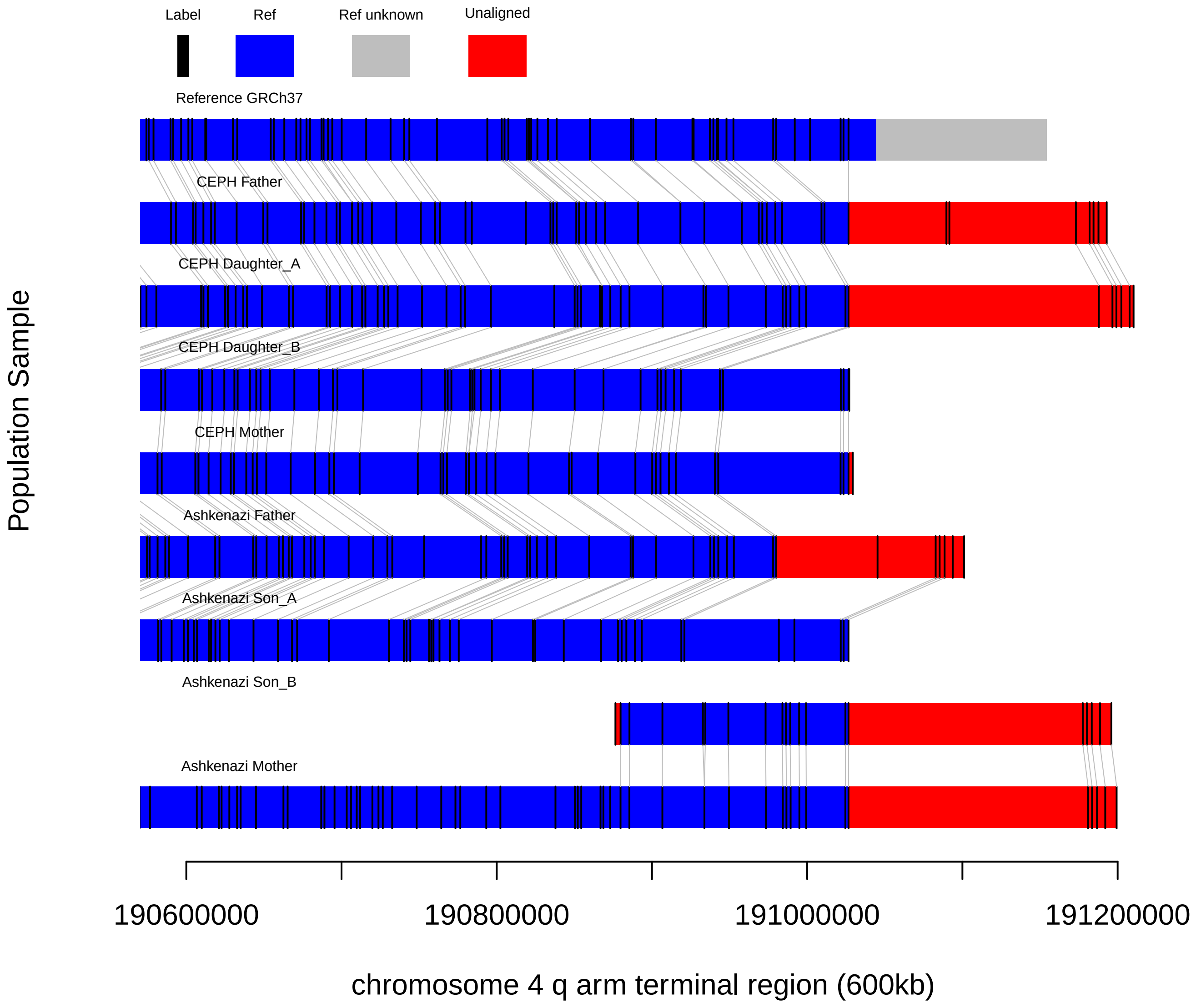

Figure S3. Chromosome 4q diploid sequence in BioNano assembly from two trios.
"_A" and "_B" indicate diploid sequence. Each contig is drawn as a color block, where the color indicates homology, and overlapping colors indicate homology to two sources. The enzyme recognition sites (labels) are marked as black bars. The homology sequence is drawn with the same color. Blue is for human reference or its homology, grey for reference unknown sequence. The remaining unaligned regions are all colored with red. The gray line connections between labels indicate they are aligned to each other(see Methods).

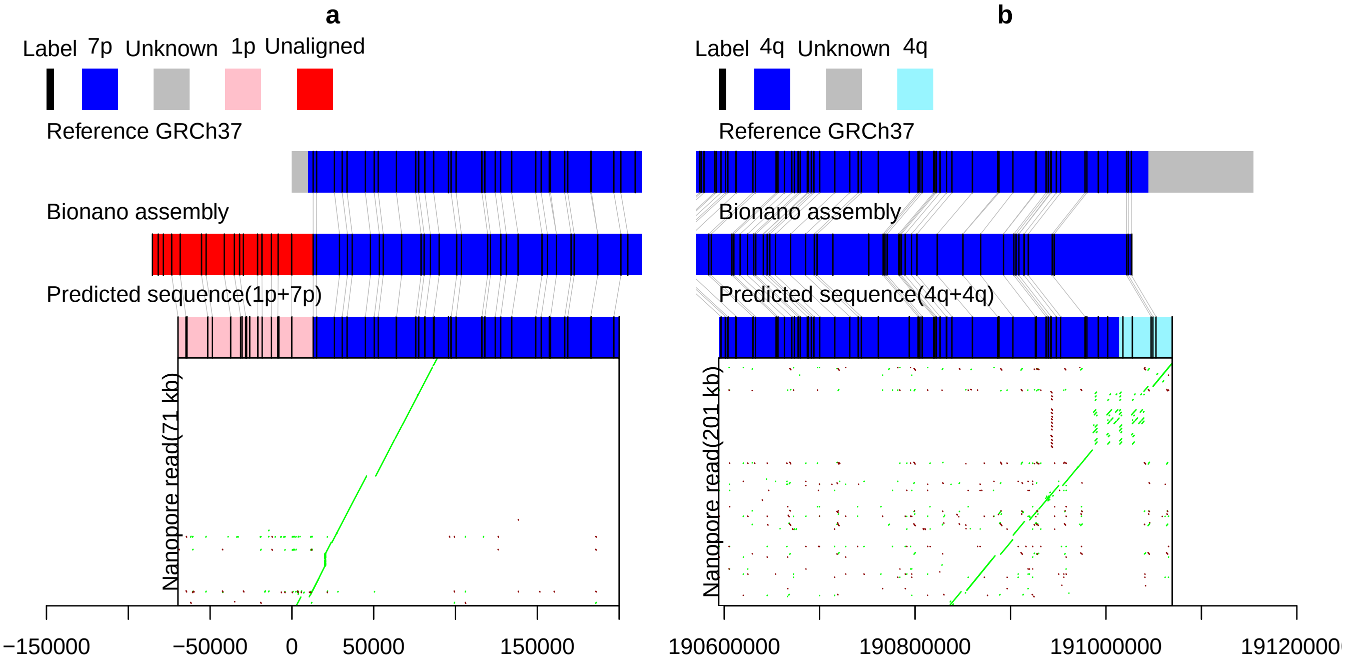

Figure S4. **Comparison of two terminals from bionano assembly and nanopore reads.**
__a__ and __b__ are for 7p and 4q, respectively. Reference, bionano assembly in NA12878 and predicted extension sequence(see Methods) are showed as coloured rectangle in the middle. In silico bionano enzyme recognition sites (labels) are showed as vertical black line. The grey lines between labels indicate they are matched. The dotplots of nanopore read to extension sequence are shown at the bottom. Green and red are forward and reverse alignment, respectively.

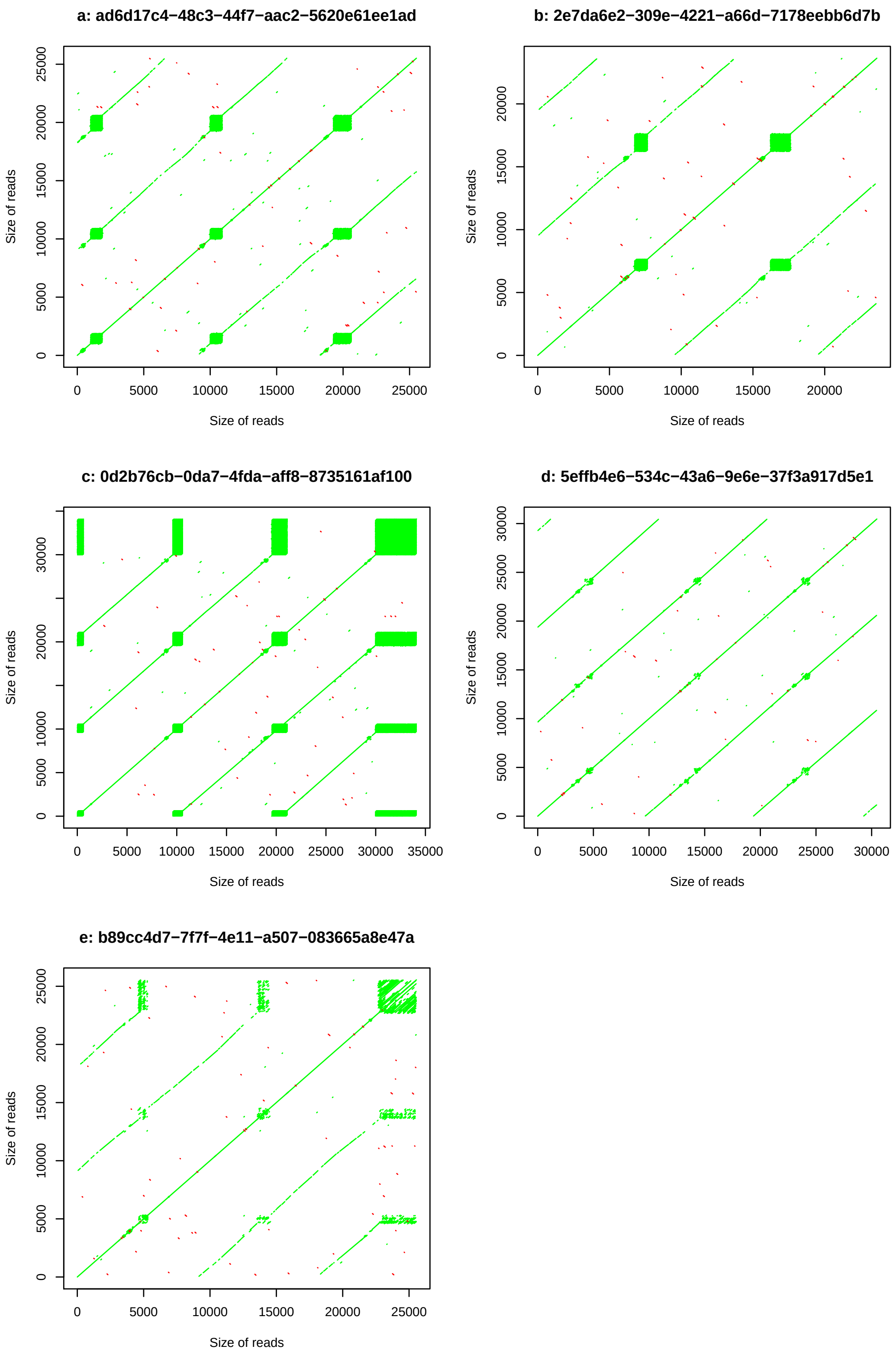

Figure S5. **Self dotplots for five nanopore reads.**
Green and red mean forward and reverse alignment, respectively. The alignments were done by YASS[1]

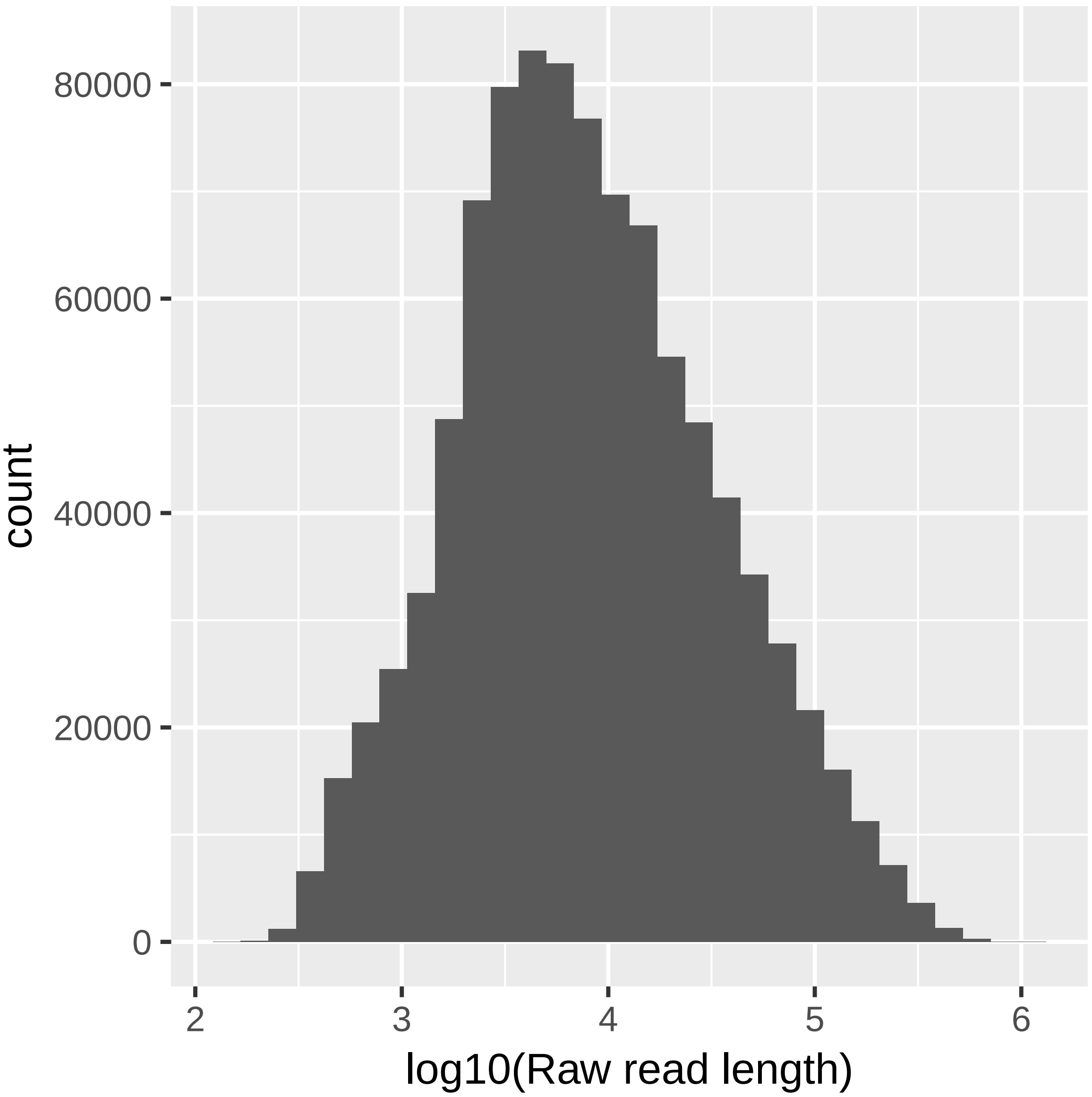

Figure S6. Histogram of raw nanopore read length.

References

1. Noé, L. & Kucherov, G. Yass: enhancing the sensitivity of dna similarity search. *Nucleic acids research* **33**, W540–W543 (2005).
